# Feasibility Study of Diffusion Welding of Microcomposite Copper-Niobium Conductors

**DOI:** 10.3390/ma19132931

**Published:** 2026-07-07

**Authors:** Nikolaj Višniakov, Paulius Beinoras, Oleksandr Kapustynskyi

**Affiliations:** 1 Institute of Mechanical Science, Vilnius Gediminas Technical University, Plytines g. 25, LT-10105 Vilnius, Lithuania; 2 Department of Mechanics and Material Engineering, Vilnius Gediminas Technical University, Plytines g. 25, LT-10105 Vilnius, Lithuania; paulius.beinoras@vilniustech.lt (P.B.); o.kapustynskyi@vilniustech.lt (O.K.)

**Keywords:** diffusion welding, glow discharge, diffusion bonding, microcomposite conductor, interlayer

## Abstract

The present research provides the results of an experimental study of diffusion-welded joints between Cu–Nb microcomposite conductors designed for potential application in electrical connections for magnetic systems. The joints were produced in the solid state using uniaxial diffusion bonding and glow-discharge diffusion welding with metal foil interlayers, which made it possible to avoid remelting the Cu–Nb conductor and limit the degradation of its filamentary microstructure. The effect of the interlayer materials on the microstructure, as well as on the mechanical and electrical properties of the joints, was evaluated. Among the configurations studied, the Cu–Ti–Cu interlayer provided the best combination of properties, with a tensile strength of 400 MPa, a yield strength of 220 MPa, an elongation of 2.5%, and an electrical conductivity of 51.6% IACS. Compared to the initial conductivity of the conductor (65.1% IACS), this corresponds to a reduction in conductivity of approximately 20.7%. The results demonstrate a clear trade-off between mechanical and electrical characteristics when using interlayers containing titanium. Thus, diffusion bonding using a glow discharge and foil interlayers is considered a promising approach for Cu–Nb conductor joints that are not directly exposed to the maximum magnetic and tensile forces generated in high-power solenoids.

## 1. Introduction

The development of modern equipment and technology requires advanced metals, alloys, and metal composites with high mechanical and operational performance under various temperatures and loading conditions; resistance to corrosion and erosion in aggressive environments; and stable physical properties when exposed to electromagnetic fields, electric currents, and radiation [1,2,3,4]. Such materials include superhard alloys, refractory and active metals, and metal matrix composites [5].

Many of these materials are expensive, exhibit limited ductility, are mutually insoluble, and have high melting temperatures [6], making their joining a significant technological challenge. Nevertheless, existing theoretical and experimental studies indicate that high-quality bonding of such materials, including dissimilar combinations, can be achieved by solid-state pressure welding [7,8,9,10].

However, the success of these approaches is strongly influenced by the physicochemical compatibility of the materials and the selected welding technique. In this context, the present study investigates the joining of Cu–Nb microcomposite conductors using diffusion welding. This work continues a series of studies conducted at VilniusTech on joining such conductors using thermite, laser, electron beam, magnetic pulse, and resistance welding techniques, which demonstrated feasibility but also revealed limitations in joint quality and applicability [11,12,13,14,15,16,17]. At present, no reliable experimental data confirm the effectiveness of diffusion welding for Cu–Nb microcomposites, although theoretical considerations suggest that joining such dissimilar metals is possible [7,18,19,20,21].

Diffusion welding is a versatile solid-state process capable of joining materials that form solid solutions, intermetallic phases, or are mutually insoluble [16,21,22,23,24,25,26]. However, when welding dissimilar metals with significantly different properties, deformation tends to occur primarily in the softer material. Additionally, the process temperature (below the solidus) may be insufficient to activate the surface of refractory materials. Under such conditions, even if thermodynamic criteria are satisfied, bonding may not occur due to insufficient formation of active centers at the interface [18,27,28]. Therefore, metal pairs with large differences in melting temperature and physical properties may remain difficult to join in the solid state. Consequently, the feasibility of diffusion welding of Cu–Nb microcomposite conductors requires experimental validation.

Due to the very limited and fragmented published information about the diffusion joining of Cu–Nb microcomposite conductors, the following sections review and summarize the diffusion welding mechanisms and technological factors that directly justify the chosen experimental strategy and help interpreting the obtained results.

### 1.1. Overview of the Main Conditions and Characteristics of Pressure Welding Methods

**Figure 1:**
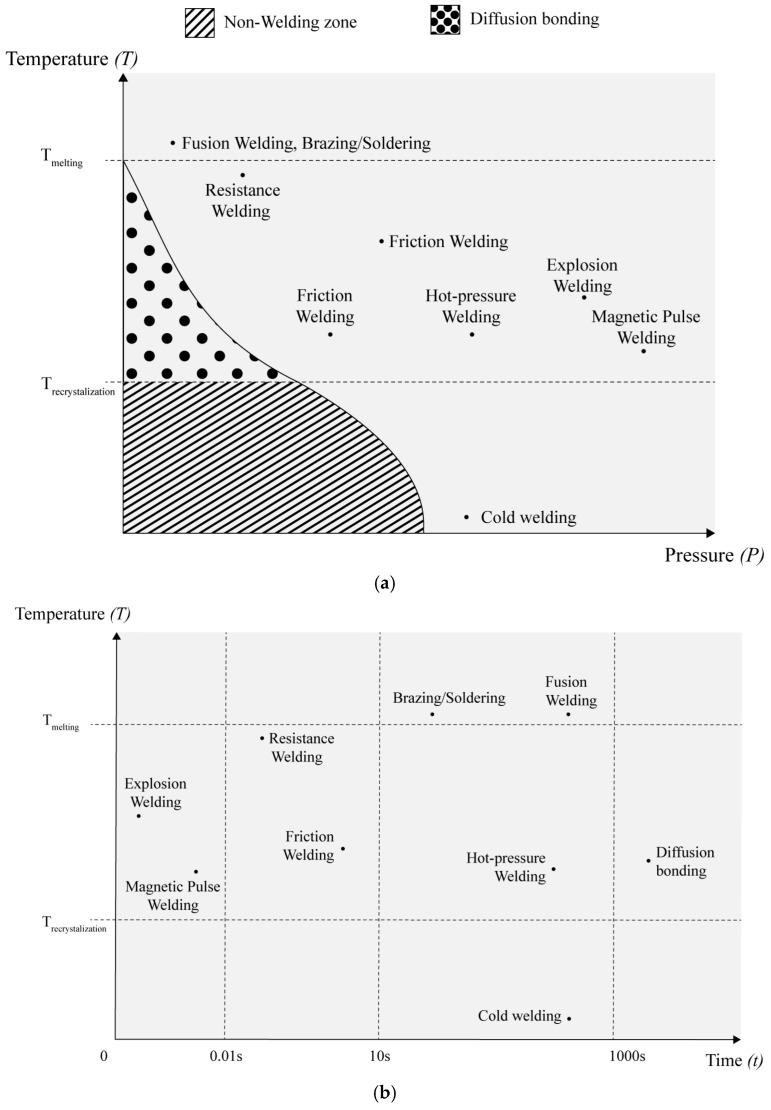
Schematic of welding pressure, temperature, and bonding time combinations for different solid-state welding processes: (**a**) excellent metallurgical bond and mechanical properties due to the absence of melting [31]; (**b**) smaller size heat-affected zone (HAZ) due to the focused heat input [31].

All pressure welding methods can be classified into three groups: low-intensity force with free deformation (diffusion welding), medium-intensity force with forced deformation (e.g., cold welding, friction welding, rolling), and high-intensity impulse force (e.g., explosion welding, magnetic pulse welding) [21,27,28,29]. During welding with low-intensity deformation, where residual deformation does not exceed approximately 10%, and the process duration may reach several tens of minutes, relaxation processes develop in the joint region. The influence of residual stresses and welding-induced deformation on joint properties is typically limited [25,26,27,28,29,30]. In contrast, during welding with medium- and high-intensity deformation, where residual deformation can reach tens of percent, and the process duration ranges from 10^−6^ s (as in explosion welding) to several seconds (as in cold welding), the influence of stresses on the properties of welded joints becomes significant [25,26,27,28,29,30]. Therefore, the deformation rate of the welded materials must be compatible with their relaxation behavior under welding conditions [21,27,28,29,30,31]. The corresponding qualitative process maps are summarized in Figure 1 [31].

Dissimilar metals with limited mutual solubility can be welded using methods from the first group (at low temperatures), selected methods from the second group, and all methods from the third group [21,22]. The applicability of these methods depends on the thermomechanical conditions of the welding process [21,22,31], as summarized schematically in Figure 1. The conditions for obtaining a high-quality pressure weld of dissimilar materials with limited solubility can be expressed as follows [7]:

t_w_ ≥ t_b_ ≥ t_r_ (1)

t_l_ ≥ t_c_ + t_x_ (2)

where t_w_ is the duration of force application for a given welding method; tb is the duration of bonding of the contact surfaces over the entire joint area (i.e., formation of bonds between all atoms across the interface); t_r_ is the duration of stress relaxation in the contact zone; t_l_ is the duration of the latent period associated with the formation of a thermodynamically stable nucleus of a new phase in the joint region; t_c_ is the duration of metal contact at a constant temperature exceeding 0.5 T_m_ (where T_m_ is the melting temperature); and t_x_ is the duration of cooling of the metal in the contact region after welding to a temperature ≤ T_m_ [7].

In pressure welding, the joint is formed by the combined plastic deformation of the materials, i.e., mechanical activation of the contact surfaces at the interface. In this case, heating (at a temperature below the melting points of both materials) acts as an accompanying factor that promotes the development of surface interaction processes (thermal activation) [27,28,29]. This stage involves several important physical processes, including the destruction of oxide layers (surface cleaning) and an increase in the energy of surface atoms to a level at which chemical interactions and the formation of interatomic bonds become possible [27,28,29]. The bonding process in the presence of heating does not terminate at the initial formation of interatomic bonds, as diffusion-driven atomic transport across the interface continues, leading to the development of the volume interaction stage [18].

In pressure welding, the kinetics of physical contact formation, surface activation, volumetric interaction, and stress relaxation are primarily governed by variations in the deformation rate of the welded materials within the contact zone during the welding cycle, i.e., the deformation pattern [27,28,29].

**Figure 2:**
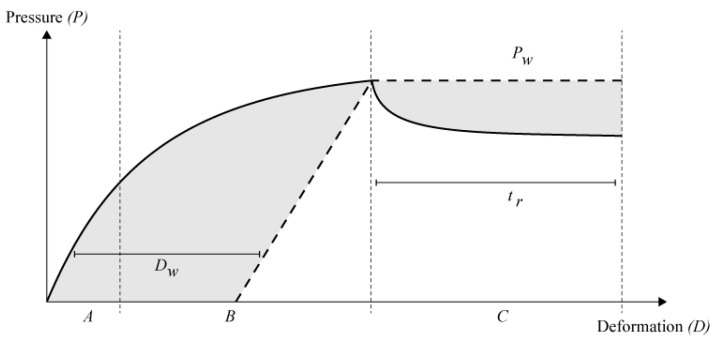
Scheme of deformation during pressure welding: t_r_—relaxation time, D_w_—cumulated deformation; P—applied pressure; P_w_—cumulated pressure; A—macro elastic stage; B—plastic deformation stage; C—relaxation stage [32].

In pressure welding practice, three main process schemes are distinguished, differing in the nature of the applied load or stress during the welding cycle. In the first scheme, the load and plastic deformation occur at a controlled rate (forced deformation scheme, Figure 2). This approach is most commonly applied in cold welding, although it can also be used in diffusion welding [21]. When welding according to the forced deformation scheme, the welding cycle may be limited to the macroelastic stage (Stage A), or interrupted at any point during the plastic deformation stage (Stage B) or the stress relief and relaxation stage (Stage C), where no significant increase in plastic deformation occurs, based on the general pressure-welding principles described in Ref. [32]. Consequently, welding with forced deformation is typically carried out under mechanical stresses that exceed the yield strength of the materials being joined.

Another effective method for intensifying the formation of physical contact between the surfaces being welded is the cyclic variation in welding pressure. In this approach, the process consists of alternating stages: (1) compression at a specified pressure P_1_ with a holding time t_1_, followed by unloading for a time t_2_; and (2) repeated compression at P_2_ = P_1_ with t_3_ = t_1_, followed by unloading for a duration of t_4_ = t_2_, and so on. Such a loading scheme significantly increases the accumulated plastic deformation of the material compared to static loading conditions. This process can be implemented in magnetic pulse welding and other high-rate deformation-based joining methods, where cyclic or pulsed loading promotes enhanced interfacial contact and bonding [32].

The simplest and most commonly applied loading scheme in pressure welding is a constant load below the yield strength. Under these conditions, the processes occurring in the welded materials are analogous to creep deformation. This approach is characteristic of diffusion welding and is typically carried out according to the free-deformation scheme [21,25,26].

The mechanisms governing the diffusion bonding process include both deformation- and diffusion-controlled processes. The deformation mechanisms comprise plastic deformation and creep, with creep playing a particularly important role in facilitating the closure of interfacial microvoids [21,25,26]. The diffusion mechanisms include surface, volume, interface, and grain boundary diffusion, all of which contribute to the formation and growth of metallurgical bonds across the interface [24,33].

The bonding interface forms through the deformation of surface asperities when the components to be joined are pressed together at relatively low pressure under elevated temperature. The applied pressure may be uniaxial or isostatic [21,25,26].

In uniaxial pressing, relatively low pressures (typically 3–30 MPa) are used to prevent macroscopic deformation of the components (generally limited to a few percent). Consequently, this process requires a high-quality surface finish of the mating surfaces, since the contribution of plastic deformation to bonding is limited. Typically, an average surface roughness (Ra) better than 0.4 µm is recommended. In addition, the surfaces must be thoroughly cleaned to minimize contamination and ensure effective bonding [21,23].

In hot isostatic pressing (HIP), significantly higher pressures (typically 100–200 MPa) can be applied. As a result, surface finish requirements are less stringent, and roughness values of approximately 0.8 µm (Ra) or higher may be acceptable. An additional advantage of this method is that uniform gas pressurization enables the bonding of components with complex geometries, in contrast to the relatively simple butt or lap joints typically achievable with uniaxial pressing [29,30].

According to ISO/TR 25901-3 [34], diffusion welding is a pressure welding method in which the joint is formed by plastic deformation below the melting point (i.e., in the solid state) with relatively small residual plastic deformation. This approach minimizes problems associated with liquation, cracking, and residual stresses, allowing joints to be produced without subsequent machining [21,25,26].

Heating during diffusion welding can be achieved using various heat sources, including induction heating, radiation heating, electron beam heating, resistive (Joule) heating, glow discharge heating, and heating in molten-salt media [21]. The contact between the parts to be joined may be established directly or through interlayers.

When pressure welding with heating is carried out in a vacuum, the metal surfaces are protected from oxidation. They can also be cleaned through desorption, sublimation, and diffusion processes into the bulk material. This promotes the formation of a metallic bond across the contact interface [21,25,26]. Therefore, diffusion welding is most commonly performed in vacuum or in controlled atmospheres consisting of protective or reducing gases and their mixtures. For materials with low oxygen affinity, the process may also be performed in air under appropriate conditions [21].

Additional methods for intensifying diffusion processes in the welding zone include applying tensile stresses perpendicular to the welding pressure, and using ultrasonic vibrations, impact loading, or ionizing radiation (e.g., electron beam irradiation) [21,35]. Each of these approaches can enhance bonding; however, they require complex, specialized, and expensive equipment, particularly for processes involving cyclic or pulsating loading conditions.

To control the structure and properties of the welded joint, thermomechanical treatment may be applied either during or after welding (e.g., homogenization annealing) [21]. Before welding, the surfaces to be joined must be mechanically prepared to achieve a surface roughness (Ra) ≤ 1.25 µm. Immediately before welding, the surfaces are cleaned to remove grease, contaminants, and oxide films using electrochemical or chemical treatments (e.g., etching, degreasing, or electropolishing) [14].

An effective additional method is the use of electrical discharge cleaning directly within the working chamber before welding, which further improves surface activation and bonding conditions [36,37].

In practice, an effective method for intensifying the diffusion bonding process and addressing a wide range of joining challenges is the use of interlayers (e.g., foils, powders, galvanic or sprayed coatings), which may be fusible or non-fusible [7,16]. The interlayer material is typically selected such that its diffusion rate into the base materials is higher than that of the base metal elements into the interlayer, thereby promoting interfacial bonding and homogenization [7,16].

In diffusion bonding, low-melting-point interlayers (in the form of coatings, foils, or powder mixtures) can be used to introduce a transient liquid phase at the interface. The presence of a liquid phase reduces the required deformation load and bonding temperature, which is particularly advantageous when joining difficult-to-deform, heat-resistant, or compositionally complex materials [38,39]. Materials used for such interlayers are typically based on brazing alloys or reactive compounds that interact with oxide films and the substrate surface, thereby facilitating their removal or redistribution from the contact zone during bonding [38,39].

In many cases, the transient liquid phase promotes surface cleaning (through separation, dispersion, and dissolution of oxide films, as well as their displacement from the contact zone), similarly to brazing, and enhances wetting of the joined surfaces [38,39]. However, a potential disadvantage of this approach is that the interlayer composition may influence joint properties. In some cases, the joint strength may be lower than that of the base materials, approaching that of brazed joints [16,38,39].

Another approach to joint formation involves using a liquid interlayer formed at the eutectic temperature due to interactions between the interlayer material and the base materials in the contact region. In such systems, the interface between the base material and the eutectic interlayer becomes diffuse, and the resulting microstructure and physical, chemical, and mechanical properties are governed by the combined characteristics of both materials [38,39].

The eutectic alloy formed between the base materials and the interlayer exhibits properties intermediate between those of the constituent materials, which can improve bonding but may also influence the final joint performance depending on the phase composition and distribution [38,39].

When joining dissimilar or composite materials, non-melting interlayers made from highly active, ductile metallic materials are often used, including gold, silver, nickel, copper, and aluminum. These interlayers may be introduced into the joint region as foils, wires, or powders. Thin interlayers may also be applied directly to the joining surfaces as coatings produced by electroplating, chemical deposition, or vacuum deposition methods [16,21].

The use of intermediate layers reduces the bonding temperature and facilitates deformation of surface asperities, thereby promoting contact formation and surface activation. When welding materials prone to intermetallic phase formation at the bonding temperature, the use of special barrier interlayers becomes necessary to suppress their formation in the joint region [16,38]. In such cases, foil interlayers are commonly employed to limit the formation of brittle intermetallic compounds at the interface. The mechanical performance of the joint is then strongly influenced by the properties of the interlayer material. To ensure sufficient load-bearing capacity, relatively thick foil interlayers (typically 100–500 µm) are used, including single- and multilayer configurations. These interlayers are selected to be compatible with the materials being joined and to act as diffusion barriers [16,38]. Interlayers made of ductile metals (e.g., Ni, Cu, Ti, Ag) are frequently used to enhance plastic deformation, particularly when joining materials with high rigidity or limited deformability within the bonding temperature range (e.g., heat-resistant alloys or poorly matched surfaces) [16]. A similar effect can be achieved using powder-based interlayers, which are preformed as inserts (e.g., tapes or preforms) from industrial powders (typically 50–100 µm particle size) or ultrafine powders (≤1 µm). These interlayers improve contact conditions and promote bonding by enhancing deformation and diffusion. Thicker, ductile interlayers are also required when significant differences in mechanical properties or surface geometry hinder direct bonding between the materials [16].

A similar effect can be achieved using powder-based interlayers, which are preformed as inserts (e.g., tapes or preforms) from industrial powders (e.g., Cu, Ni, Ti powders with particle sizes of 50–100 µm) or ultrafine powders (≤1 µm). Thicker, ductile interlayers are often required when joining materials with significantly different coefficients of thermal expansion. In such cases, the interlayers accommodate residual stresses and reduce the risk of joint failure during cooling [16].

Thin coatings (typically 5–10 µm) applied by electroplating, chemical deposition, or vacuum deposition methods to one or both joining surfaces can also serve as interlayers. The quality of joints obtained with electroplated or sprayed coatings strongly depends on their adhesion to the base material, which, in turn, is governed by the quality of surface preparation [21].

To achieve strong adhesion, the microstructure and physicochemical properties of the base material and the deposited layer must be compatible. Interdiffusion between the coating and the substrate further enhances adhesion, particularly when it leads to the formation of a solid solution at the interface [18].

Electrolytically deposited coatings (group C25D) may differentiate from bulk materials in terms of electrical conductivity, hardness, internal stresses, magnetic properties, and microstructure. Such coatings typically have a fibrous, filamentary, or columnar microstructure, which can affect their performance during welding. Such coatings can also protect the materials being welded, especially those containing oxygen-reactive elements (e.g., Cr and Al), from oxidation during heating. Copper- or silver-based coatings are typically used for this purpose [7,8,25,26].

To prevent adhesion between the workpieces and the tooling, barrier coatings are often employed. These coatings (group C09D) may include materials based on aluminum oxide or mixtures containing organic binders such as acrylic resins or polyvinyl alcohol, typically applied using suitable solvents (e.g., acetone or other organic solvents) [21].

In addition, insulating layers such as mica, alumina (alundum), boron carbonitride, ceramics, heat-resistant fabrics, and glass fiber materials may be used to provide thermal and electrical isolation during the welding process [21].

Depending on the materials being welded, the vacuum level in the working chamber is typically set to 1.3 × 10^−4^ Pa. When welding copper-based alloys, the requirements for residual pressure are relatively less stringent. However, the presence of active elements such as chromium, aluminum, titanium, and tungsten necessitates a significant reduction in residual pressure to prevent oxidation [21].

Controlled atmospheres commonly used in diffusion welding include argon or helium, purified and dried hydrogen, or mixtures of nitrogen with 6–8% hydrogen [21,25,26,28].

The composition of salt baths used in diffusion welding is determined by the required process temperature. For example, NaCl is used at temperatures of 1123–1143 K, BaCl_2_ at 1273–1423 K, and mixtures such as 70% BaCl_2_ + 30% KCl at 973–1223 K [21].

Another approach to diffusion welding involves hybrid processes that are technologically simpler and more cost-effective than conventional methods. One of the most efficient heating approaches in such systems is the use of distributed gas-discharge plasma generated by a normal glow discharge, operating in inert or reactive gas environments at medium pressures (typically 0.1–100 kPa) [37].

In this process, welding is carried out under reduced-pressure conditions in the presence of an active gas atmosphere (commonly argon, hydrogen, or their mixtures), which sustains the glow discharge. The plasma contributes to surface activation by sputtering and removing oxide films, thereby reducing the force required to achieve effective physical contact between the joining surfaces [37].

The use of glow discharge as a heat source for diffusion bonding is effective due to the wide range of controllable process parameters, high adaptability to varying component geometries, and high energy efficiency (typically 0.7–0.85), as well as the relatively simple design and lower cost of the equipment [36,37].

**Figure 3:**
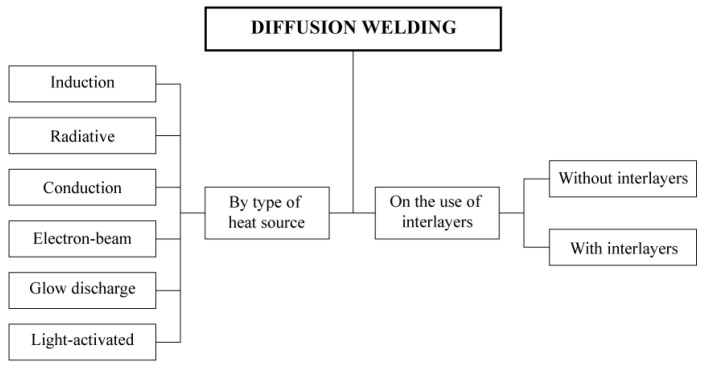
Classification of diffusion welding processes [41].

According to ISO 4063 [40], diffusion welding is designated as process 45 (DFW). However, the standard ISO 4063 does not provide a detailed classification of all currently known variants of diffusion bonding. Based on the above considerations, diffusion bonding processes may be classified according to their principal technological characteristics: by bonding mechanism (solid-state diffusion bonding and transient liquid phase (TLP) or eutectic diffusion bonding); by pressure application method (uniaxial pressing, hot isostatic pressing (HIP), differential gas pressure); by the type of heat source (induction, radiative heating using resistive heaters, electron beam, laser, electrical resistance, glow discharge, furnace heating); by the use of interlayers (with or without interlayers); and by the type of working atmosphere (vacuum or protective atmosphere) (Figure 3, Table 1).

Currently, diffusion bonding can be applied to join a wide range of structural metals and their alloys, including both similar and dissimilar material combinations with significantly different properties. Technologies for joining a large number of dissimilar material pairs have been developed, demonstrating the versatility of this process [7,21].

When joining identical materials by diffusion bonding, the microstructure and properties of the joint can closely approach those of the base material. In dissimilar-material systems, mechanical properties in the contact zone are influenced by solid-state interactions, leading to the formation of a transition zone with characteristics intermediate between those of the base materials [21]. This process involves diffusion-driven homogenization, grain growth, and the reduction in interfacial defects, which can result in high joint strength under appropriate process conditions.

Composite materials reinforced with dispersed particles or fibers can also be joined by diffusion bonding without significantly disturbing the reinforcing phase, while largely preserving the original microstructure and mechanical performance of the material [42].

Despite significant progress in diffusion bonding, several unresolved challenges remain that are difficult—and in some cases impossible—to address within the framework of conventional approaches. This is particularly relevant for the joining of magnetic and amorphous alloys, piezoelectric and optical ceramics, as well as semiconductor and composite materials, where exposure to temperatures above approximately 0.7 T_m_ (melting temperature) and welding pressures exceeding 0.8 R_e_ (yield strength) can lead to irreversible changes in the material properties or even structural degradation. Therefore, a key direction in the development of advanced diffusion bonding technologies is to intensify the bonding process, enabling the formation of high-quality joints at reduced temperatures (below approximately 0.5 T_m_) and under pressure conditions that minimize or eliminate macroplastic deformation in the contact zone [23].

These research directions are highly relevant for addressing critical challenges in the reliable joining of advanced materials, including micro- and nanostructured composite conductors, magnetic induction, and superconducting systems [3,4,32].

### 1.2. Cu–Nb Microcomposite Conductors: Specific Scientific Challenges for Diffusion Bonding

The principles of diffusion bonding described above are applicable to a wide range of combinations of homogeneous and dissimilar materials. However, nanoscale filamentary Cu–Nb conductors present further scientific and technological challenges. The objective in this case is not limited to the formation of a metallurgical bond. The welding conditions should also preserve the nanoscale filamentary architecture, which provides a combination of high mechanical strength and electrical conductivity of conductor. Overheating and excessive deformation can lead to agglomeration or spheroidization of the Nb filaments, whereas insufficient activation can hinder the formation of an adequate bond due to the limited mutual solubility of Cu and Nb and the presence of surface oxides. Therefore, diffusion bonding of Cu–Nb microcomposite conductors requires a particularly narrow range of process parameters.

Pure copper (Cu) and niobium (Nb) differ significantly in crystal structure and melting temperature: Cu has a face-centered cubic (FCC) structure, whereas Nb has a body-centered cubic (BCC) structure. Copper melts at approximately 1357 K, while niobium melts at about 2741 K. Consequently, the characteristic diffusion welding temperature for copper corresponds to a much lower homologous temperature relative to niobium, i.e., T/T_m_ (Cu) ≫ T/T_m_ (Nb) [1,43,44].

According to the Cu–Nb phase diagram, copper and niobium exhibit very limited mutual solubility in the solid state under equilibrium conditions, with approximately 0.1–0.2 at.% Nb in Cu and about 1.2 at.% Cu in Nb [4,45,46,47,48]. This low mutual solubility is a key factor enabling the formation of high-strength, high-conductivity Cu–Nb composite wires, as it suppresses the formation of intermetallic compounds and promotes a microstructure consisting of distinct copper and niobium phases (Table 2) [1,2,44,49,50].

Cu–Nb (copper–niobium) microcomposites represent a class of advanced materials that combine high electrical conductivity with high mechanical strength, making them particularly suitable for applications such as conductors in high-field and pulsed magnet systems [50,51,52,53]. These materials consist of a copper matrix reinforced with high-aspect-ratio niobium filaments, which are formed through intensive thermomechanical processing, such as severe plastic deformation [52,53,54,55,56,57].

Currently, the most used methods for joining such materials include mechanical fastening (e.g., bolted connections) and soldered joints [11,23]. However, these approaches do not provide joint properties equivalent to those of the base material. They may lead to reduced mechanical strength and increased electrical resistance compared to a continuous conductor. At the same time, they are often preferred over conventional fusion welding methods, which involve melting and can significantly degrade the microcomposite structure, resulting in deterioration of both mechanical and electrical performance [12,13,14,15,16].

Alternative joining techniques, such as flash welding, upset resistance welding, laser welding, electron beam welding, and thermite welding, have been investigated as potential methods for producing joints while minimizing structural damage. However, these methods also have limitations in terms of process applicability, especially for magnet winding systems, and may result in reduced mechanical performance compared to the base microcomposite material [12,13,14,15,16].

As shown by the analysis of published scientific data and previous experimental studies on diffusion bonding of Cu and Nb, as well as available data on the oxidation behavior of these materials under diffusion bonding conditions, this method can be considered a promising, yet still insufficiently explored, approach for joining Cu–Nb microcomposite materials in the solid state [18,19,20].

As reported by Liu et al. [19], the primary challenge in diffusion bonding of Cu and Nb arises from the immiscibility of the Cu-Nb system. Neither solid solutions nor intermediate phases are formed over a wide temperature range. Consequently, interdiffusion and chemical interaction at the interface are limited, making it difficult to achieve strong metallurgical bonding without extremely high bonding temperatures approaching the melting point of copper.

This investigation was carried out on a Cu–Nb microcomposite conductor with niobium filament diameters below 15 nm—a commercially available Cu–Nb 18 wt.% A microcomposite wire with dimensions of 2.4 mm × 4.2 mm was used for the welding experiments.

The tensile strength (R_m_) of the Cu–Nb 18 wt.% conductor reaches 1100–1500 MPa, the yield strength (R_e_) is approximately 850 MPa, the relative elongation is about 4.2%, and the electrical conductivity is approximately 65% IACS [1,2,50,58,59,60,61].

**Figure 4:**
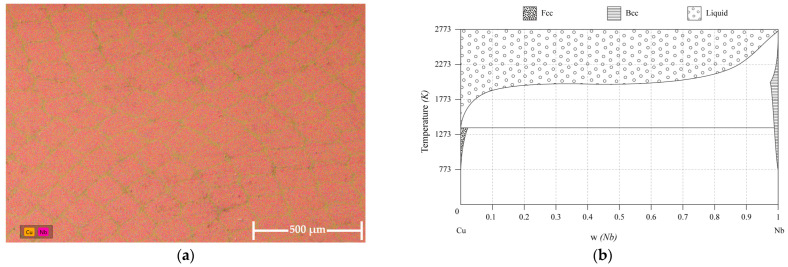
Cross-section of Cu-Nb conductor: (**a**) microstructure, ×250; (**b**) Cu-Nb phase diagram [4,45,46,47,62,63,64].

The present study focuses on segments of this conductor (Figure 4, Table 3) and their welded joints, which were produced using diffusion bonding.

### 1.3. Effects of Surface Oxides

In some cases, processes occurring in the bonding zone proceed slowly due to the presence of oxide layers and the limited mobility of elements. Native oxides on material surfaces can act as diffusion barriers. In Cu–Nb diffusion bonding, the presence of oxygen can significantly influence the bonding process under certain conditions [65,66].

**Figure 5:**
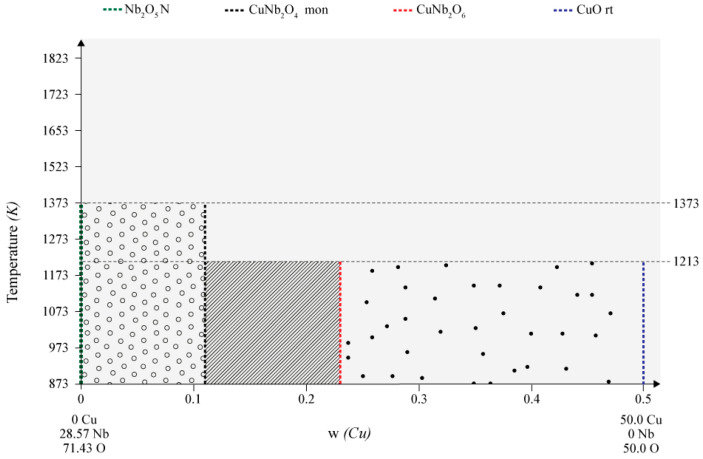
Cu-Nb-O ternary phase diagram (isopleth) [65].

**Figure 6:**
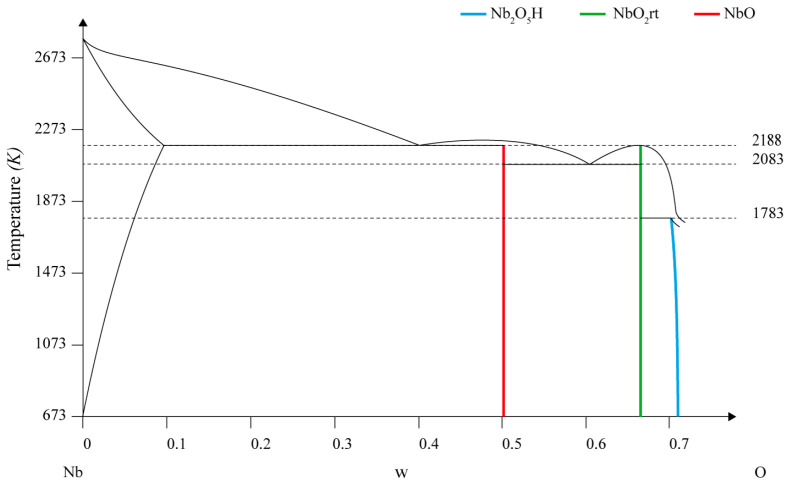
Nb-O binary phase diagram [21].

For example, oxide compounds such as CuNb_3_O_8_ are thermally unstable and decompose at approximately 1213 K (Figure 5) [21,65,66]. In addition, increasing oxygen content in niobium leads to a reduction in its melting (liquidus) temperature and promotes the formation of various intermediate oxide phases (Figure 6) [21,65,66].

**Figure 7:**
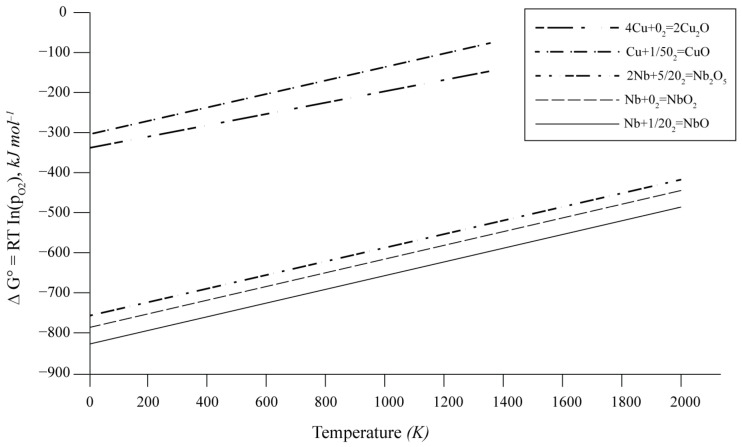
Ellingham diagram for copper and niobium oxides [66].

At the same time, niobium has a strong affinity for oxygen and can reduce copper oxides to metallic copper because of its greater chemical reactivity, as summarized in Figure 7 [66]. Under appropriate furnace conditions (controlled atmosphere and temperature), oxide layers on niobium surfaces can be reduced. Furthermore, a thin native oxide layer (on the order of ~2 nm) can reform on niobium surfaces upon exposure to air and moisture; however, such thin layers generally do not significantly hinder diffusion bonding processes [21,65,66].

Due to the presence of oxide layers and the limited diffusion mobility of niobium, it is often necessary to remove surface oxides prior to bonding. This can be achieved by mechanical cleaning, chemical etching, or the use of additional functional materials (activators) designed to remove or disrupt oxide films in the contact zone (e.g., paraffin, ammonium fluoride, or fluxes) [7,21].

The aim of this study was to evaluate the possibility of using diffusion welding to join Cu–Nb microcomposite conductors without remelting the conductor or significantly degrading its filamentary microstructure. Diffusion bonding using a uniaxial pressure and glow discharge were investigated, with selected metal foils used as interlayers. To assess the potential applicability of the proposed joining method for electrical terminal connections in magnetic systems, the microstructure, element distribution, electrical conductivity, and mechanical properties of the resulting joints were analyzed.

## 2. Methodological Basis for the Selection of Diffusion-Welding Methods and Process Parameters

The references cited in this subsection are used to provide a methodological basis for the selection of the experimental welding methods and process parameters, rather than to present a general review of previous studies. Accordingly, the process parameters were selected by combining established principles of diffusion bonding with the specifics of the Cu–Nb microcomposite conductor. The selected conditions were intended to ensure sufficient activation of the interphase boundary while avoiding local remelting, excessive plastic deformation, and degradation of the nanoscale filamentary Nb structure.

The selection of the welding method and process variables was determined based on the following factors: (i) the formation of a solid-state continuous joint without remelting the Cu–Nb conductor; (ii) the ability to preserve the filamentary microstructure outside the localized interaction zone; (iii) sufficient activation of the surfaces to be welded and the formation of a diffusion layer of the target thickness of approximately 5–10 μm; (iv) limitation of macroscopic deformation and interlayer extrusion; (v) prevention of significant interphase voids and excessive coarsening or spheroidization of Nb; and (vi) maintenance of an acceptable balance between mechanical strength and electrical conductivity. The selected processing conditions should therefore be considered as preliminarily acceptable ranges rather than fully optimized parameters.

Taking into account the properties of the materials being joined and the characteristics of different welding approaches, several diffusion bonding methods can be considered suitable for joining Cu–Nb microcomposite conductors with relatively small cross-sections (~10 mm^2^). Among the available techniques, the most practical and feasible methods include uniaxial press diffusion bonding (performed in a furnace or using induction heating), resistance-based diffusion bonding (MFDC), and glow discharge diffusion bonding [7,21].

The feasibility of joining Cu–Nb conductors using resistance-based (MFDC) diffusion bonding has already been investigated in detail in previous studies [16]. Therefore, the present work focuses on two alternative methods—uniaxial press diffusion bonding and glow-discharge diffusion bonding—that have not yet been explored for this specific application and are of particular scientific interest.

For practical feasibility studies, uniaxial press diffusion welding and glow discharge diffusion welding processes were selected, as both methods allow for solid-state welding with limited macroscopic deformation. Glow discharge welding was additionally considered due to the potential for plasma-induced surface cleaning and activation. Uniaxial press diffusion welding can be applied for joining Cu–Nb conductors due to a uniform, controlled heating cycle, minimal deformation and lower thermal stresses, proper vacuum shielding.

**Figure 8:**
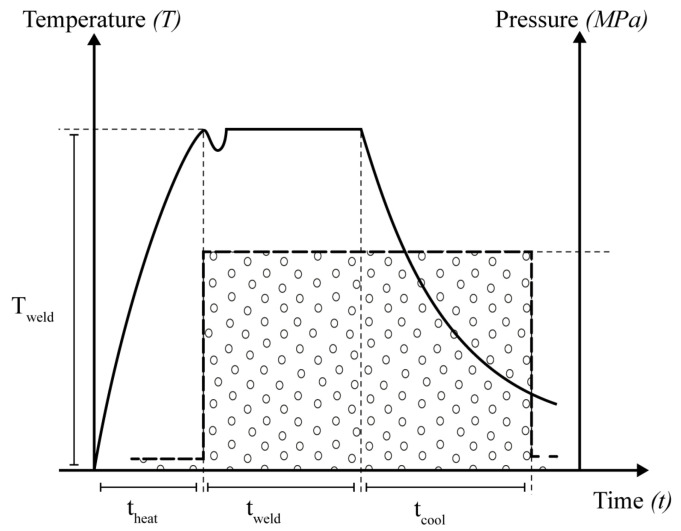
Temperature-pressure-time curve during diffusion welding [35].

The parameters of diffusion bonding depend on the physicochemical properties of the materials being joined and therefore vary for each material combination. In diffusion bonding processes operating under free deformation conditions, the primary parameters influencing joint strength include the bonding temperature T, applied pressure p, holding time t, or the degree of residual deformation ε, as well as the characteristics of the working atmosphere (Figure 8) [21,35].

Experimental studies have shown that diffusion bonding under free-deformation conditions is typically carried out within specific parameter ranges. The bonding temperature is generally selected within the range of (0.7–0.8)T_m_, and in some cases may be reduced to approximately 0.5T_m_ (based on the most fusible component of the material system). The applied pressure is typically in the range of (0.5–0.9)R_e_ (yield strength) [21,25,26].

The holding time, depending on temperature, pressure, allowable residual deformation, and surface cleanliness, can range from several seconds to several hours, typically 60–7200 s. The recommended surface roughness lies in the range R_a_ = 1.6–10 µm, while the vacuum level is typically within 1.33 × 10^−3^ to 1.33 × 10^−6^ Pa [7,21].

Heating and cooling rates depend on the heat source and are often not strictly controlled. However, when welding similar materials, it is advisable to limit the cooling rate to approximately 10–15 °C/min. For non-ferrous and reactive metals, it is recommended to release the vacuum (depressurize the chamber) only after cooling to temperatures below approximately 333 K [21].

As the temperature increases, the required pressure and/or holding time can be reduced, since the diffusion rate increases exponentially with temperature. In uniaxial press diffusion bonding, typical applied pressures range from 5 to 14 MPa [7,8,21].

The holding time must be sufficient to ensure intimate contact between the mating surfaces and to enable adequate diffusion across the interface. At the same time, excessive holding times should be avoided, as they increase process cost and may lead to undesirable effects, such as void formation in the joint zone, changes in chemical composition due to diffusion, or the formation of brittle intermetallic phases when joining dissimilar materials [21,25,26].

It should also be noted that during diffusion bonding, materials are typically heated to 0.5–0.8 T_m_. Within this temperature range, particularly in the contact zone, significant changes in mechanical behavior occur, including a reduction in yield strength and the activation of creep processes [7,8,21].

The yield strength of metals and alloys as a function of temperature can be approximately described by Equation (3) [67,68].

(3) $$\sigma_{T\cdot T}=\sigma_{T0}{\left(1-\frac{T}{T_{m}}\right)}^{2}$$

For composite materials, this relationship becomes even more complex [69,70]:

(4) $$\sigma_{C}(T)=V_{f}\sigma_{f}+V_{m}\sigma_{m}(T)\left(1-\frac{l_{c}T}{2l}\right)$$

where σ_c_(T) is the ultimate strength of the composite at temperature T; V_f_ and V_m_ are the volume fractions of the fiber and matrix, respectively; σ_f_ is the ultimate strength of the fiber; σ_m_(T) is the ultimate strength of the matrix at temperature T; l_c_(T) is the critical fiber length at temperature T, which depends on the shear stress at the fiber–matrix interface; and l is the fiber length.

Therefore, determining the optimal diffusion bonding conditions is a labor-intensive and resource-demanding task if it relies solely on empirical selection of process parameters.

Some authors propose the application of the Theory of Inventive Problem Solving (TRIZ) as a tool for optimizing diffusion bonding processes. TRIZ is based on a system of 40 inventive principles and 39 engineering contradictions, organized in a matrix used for developing new technologies and improving existing ones. The application of TRIZ enables a systematic approach to process design and parameter optimization, facilitating the production of high-quality joints for both similar and dissimilar materials, including those joined with interlayers [71].

Another widely used approach for selecting diffusion bonding parameters is based on kinetic analysis, which considers the relationship between temperature, pressure, and time in controlling atomic diffusion and bond formation. According to classical diffusion theory, atomic transport is typically described using the Arrhenius equation and Fick’s laws [72,73,74]. In this context, the diffusion coefficient D depends exponentially on temperature and is inversely related to the activation energy of diffusion, as expressed by the Arrhenius equation [33,73,75,76,77,78]:

(5) $$D=D_{0}\exp\left(\frac{-Q}{RT}\right)$$

where D is the diffusion coefficient at temperature T (m^2^/s); D_0_ is the pre-exponential factor (m^2^/s); Q is the activation energy for diffusion (J/mol); R is the universal gas constant (8.314 J·mol^−1^·K^−1^); and T is the absolute temperature (K).

For the Cu–Nb–Ti system, diffusion coefficients for solid-state reactions between element pairs are summarized in Table 4.

Fick’s Law describes how atoms diffuse from a high-concentration area to a low-concentration area [75,76,80,81]:

(6) $$J=-D\frac{d\varphi }{dt}=\frac{-D\times A\times ∆C}{∆x},$$

where J is the diffusion flux (amount of substance per unit area per unit time, kg·m^−2^·s^−1^); D is the diffusion coefficient (m^2^/s); A is the cross-sectional area (m^2^); Δx is the diffusion distance (m); and ΔC is the concentration difference (kg·m^−3^).

The diffusion length x represents the average distance that atoms travel during diffusion over a given time. It can be approximated by the following relation [7,76,82,83,84]:

(7) $$x=M{\left(D_{t}\right)}^{\frac{1}{2}}\;,$$

where x is the diffusion length; M is a constant of proportionality (often taken as ≈1); D is the diffusion coefficient at temperature T (m^2^/s); and t is the bonding time (s).

There are also more advanced models for estimating the time required for diffusion bonding, which require detailed knowledge of material properties, sample geometry, and surface conditions [85,86,87].

For example, Li et al. [27,81] developed a model based on diffusion kinetics and Fick’s law to estimate the time required to achieve sufficient bonding. The total bonding time *t* is given by:

(8) t=∫f0f−σe/ϵ3pf−2γfr0+6γr0+2r0p2ln1f−1−f2 ΩKT1+hdhDgbδ+2ΩDvdf 

where Ω is the atomic volume (m^3^); D_gb_ is the grain boundary diffusion coefficient (m^2^/s); Dv is the volume diffusion coefficient (m^2^/s); δ, d, and h are the grain boundary width, grain size, and void height, respectively (m); r_0_ is the initial void radius or surface roughness (µm); r is the instantaneous void radius (µm), with f = (r/r_0_)^2^; γ is the surface energy (J·m^−2^); σ_e_ is the effective (von Mises) stress (MPa); ε is the effective plastic strain rate (s^−1^); k is the Boltzmann constant (1.38 × 10^−23^ J·K^−1^); p is the applied pressure (MPa); and T is the absolute temperature (K).

To ensure intimate contact, the bonding pressure must be high enough to cause plastic deformation at asperity tips and to induce creep, and is therefore usually chosen below, but close to, the yield stress at the bonding temperature; typical values are 5–15 MPa for many metals. In this work, the required pressure is approximated as [21,25,26]:

*p* ≈ (0.5–0.9)*Re*(*T*) (9)

where p is the applied bonding pressure (MPa) and R_e_(T) is the yield strength at the welding temperature (MPa).

An alternative diffusion bonding approach involves using a glow discharge plasma as a heat source, which introduces several important technological differences compared to conventional heating methods. This technique is particularly suitable for components with small cross-sections, where uniform heating using conventional methods, such as induction heating, is difficult to achieve [7].

For diffusion bonding applications requiring a stable high-temperature regime and high energy density in the heating zone, a normal direct current (DC) glow discharge is considered one of the most promising solutions [36,87,88,89,90,91,92].

The energy characteristics of the discharge, which govern the heating of the cathode (i.e., the welded components), are determined by both electrical and process parameters, including discharge current (I_d_), gas pressure (P), and the inter-electrode gap length (Lc–a). In addition, these characteristics depend on the properties of the gas medium in which the discharge is sustained. A key parameter describing the gas is the cathode potential drop (U_c)_, which varies with the gas type (e.g., He, Ar, H_2_, N_2_) [92,93].

Diffusion bonding in a glow discharge is typically carried out at a current density of 0.01–1 A/cm^2^, with relatively high electrode voltages of 600–800 V and correspondingly high electric field strengths in the discharge gap [92]. The necessary and sufficient conditions for the transition of a glow discharge into an electric arc at pressures of 1.33–13.3 kPa can be expressed as follows [92,94]:

(10) $$T_{c}\geq T_{melt}\;,$$

(11) $$I\geq I_{thr}\approx 0.25\times 10^{-3}T_{melt}\sqrt{\lambda }\;,$$

(12) $$j\geq 10^{6}-10^{7}\;A/{cm}^{2}$$

where T_c_ and T_melt_ are the cathode temperature and its melting temperature, respectively (K), I_thr_ is the threshold current for arc discharge stabilization (A), and λ is the thermal conductivity of the cathode material.

**Figure 9:**
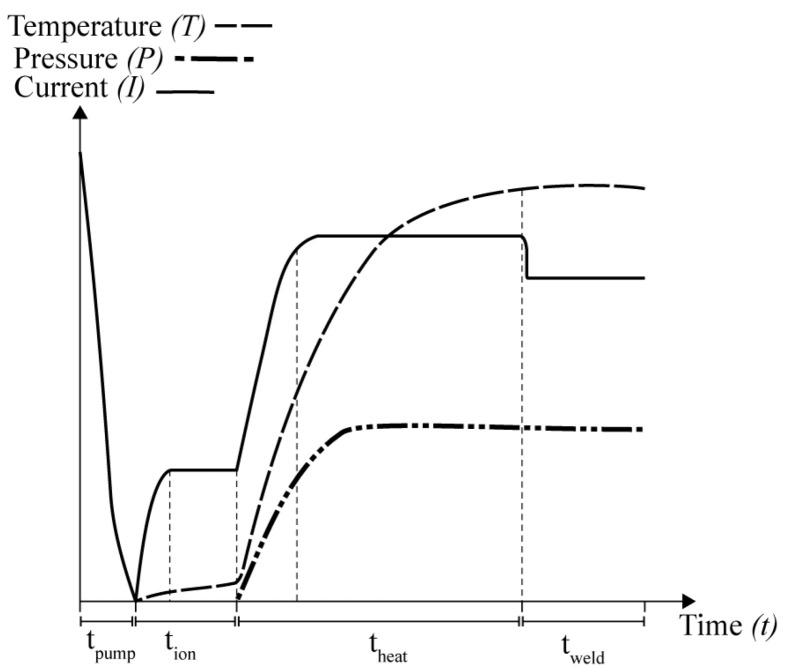
Cycle diagram of the diffusion welding process by glow discharge: I_r_—discharge current, P—gas pressure, T_c_—cathode heating temperature, t_pump_—pumping duration, t_ion_—ion cleaning time, t_heat_—duration of workpiece heating to welding temperature, t_weld_—welding time [91].

It has been established that the primary control parameters governing the heating and welding processes in glow discharge are the discharge current (I_d_), which determines the total power released in the discharge gap, and the gas pressure (P), which influences the specific energy characteristics of the discharge (Table 5, Figure 9) [90,91].

Figure 9 illustrates the characteristic cycle of the diffusion bonding process in a glow discharge, including the pumping, ion cleaning, heating, and welding stages, along with the evolution of key parameters such as discharge current, gas pressure, and cathode temperature.

The observed temperature evolution during the process is directly governed by the energy supplied by the glow discharge to the cathode. Therefore, the heating of the workpieces can be described in terms of the discharge power supplied to the cathode, which is given by [95]:

(13) $$W_{c}=I_{d}U_{c}\;,$$

The thermal energy accumulated in the sample during heating can be expressed as [95]:

(14) $$Q_{t}=\frac{c\rho VT_{c}}{t}\;,$$

where c—the specific heat of the cathode material, (kJ/kg K); ρ—the density of the material, (kg/m^3^); V—cathode volume, m^3^; t—heating time, s.

Radiation losses from the sample surface constitute a significant portion of the total heat loss. These losses depend on temperature, geometric parameters, and the cathode’s surface condition. In the absence of thermal insulation of the heated area, the radiative heat loss can be estimated using equation [95]:

(15) $$Q_{T}=\varepsilon_{0}C_{0}\left[{\left(\frac{T_{C}}{100}\right)}^{4}-{\left(\frac{T_{0}}{100}\right)}^{4}\right]S_{c}\;,$$

where C_o_ = 5.76—is the radiation constant for a black body; ε_0_ = 0.8—is the surface emissivity (typical for oxidized surfaces); T_c_—is the cathode temperature (K); T_0_ = 293 K is the temperature of the water-cooled chamber walls; and S_c_ is the lateral surface area of the cathode (m^2^).

The pressure applied during diffusion bonding with glow discharge should be sufficient to promote plastic deformation of surface micro-protrusions, thereby enhancing intimate contact between the mating surfaces [7]

(16) $$P_{CB}\approx (0.05-0.1)R_{0.2}\;,$$

where R_0.2_ is the yield strength of the softer material at the welding temperature.

Diffusion bonding of Cu–Nb systems is typically carried out at elevated temperatures (≈1073–1273 K), under moderate pressures and holding times on the order of tens of minutes, depending on the material combination and the use of interlayers (e.g., Ni, Ti, or V) to improve interfacial bonding [18,19,20].

Niobium exhibits a high affinity for oxygen and readily reacts with residual gases; therefore, in the absence of a sufficiently high vacuum, oxide formation on its surface can significantly hinder diffusion processes and degrade interfacial properties [21].

Accordingly, before bonding, the Nb surface must be carefully prepared, typically involving degreasing and chemical etching (e.g., HF + HNO_3_ solutions) to remove surface oxides and contaminants [14,20].

To overcome the immiscibility between Cu and Nb, various approaches have been explored to enhance interfacial bonding, including increasing the bonding temperature and applying surface activation or interlayer-assisted pretreatments [19].

In a study by Pan et al. [18], an interdiffusion layer approximately 36 nm thick was formed during the diffusion bonding of Cu and Nb at temperatures corresponding to 92–98% of the melting point of copper, with a holding time of 3 h.

Wagner [20] demonstrated that copper can be bonded to niobium by diffusion bonding, and that Nb diffusion into Cu occurs at processing temperatures of approximately 1123–1323 K, bonding pressures of 4–10 MPa, and holding times of 0.5–3.0 h.

Titanium (Ti) is thermodynamically compatible with copper (Cu) and niobium (Nb), but this compatibility property depends on temperature. In a study by Liu [19], a consumable Ti interlayer approximately 5 μm thick was used to facilitate interfacial bonding between Nb and immiscible Cu during diffusion bonding at 1123 K.

In the work of Karpati [96], which investigated the Cu/Nb–Ti interface using diffusion couples, it was found that at and below 1073 K the interface is free of intermetallic compounds within micrometer resolution. However, at 1098 K, Cu–Ti intermetallic phases (Ti_2_Cu, TiCu, Ti_3_Cu_4_, Ti_2_Cu_3_, TiCu_4_) were observed, forming layers thicker than 1 μm along the interface.

In another study, the phase equilibria of the Cu–Nb–Ti system at 850 °C (1123 K) were investigated using a high-efficiency diffusion-couple approach, and the TiCu_2_ phase was observed in the isothermal section [97].

In addition, studies on Cu–Ti systems [98] have shown that solid-state joining of titanium (Ti) and copper (Cu) is typically carried out below the melting point at temperatures around 850–900 °C (≈1123–1173 K), for tens of minutes, under several MPa of pressure, and often employs interlayers [99] (e.g., Ni, Nb, Zn or other alloys) to mitigate the formation of brittle Ti–Cu intermetallic compounds.

In this case, diffusion of copper into titanium is generally more pronounced than diffusion of titanium into copper, resulting in an asymmetric diffusion zone. For example, the diffusion layer on the Ti side may be approximately 2–3 times thicker than that on the Cu side [100,101]. For the Cu–Ti system, the formation of intermetallic layers with a thickness of about 5–7 μm can lead to a significant reduction in joint strength [100,101].

It should also be noted that at temperatures of approximately 1123 K and above, local interactions at Cu–Ti contact regions may lead to the formation of a transient liquid phase when the interfacial composition approaches eutectic or liquid-containing phase-field conditions [100,101]. This results in localized melting and the formation of a thin liquid layer, promoting rapid dissolution of surface asperities and adjacent solid phases.

This significantly accelerates the diffusion process in the liquid phase, where diffusion rates can be several orders of magnitude higher than in the solid state, reducing the need for high pressure to achieve plastic deformation [39]. This effect is well known in transient liquid-phase and diffusion brazing processes, where the presence of a liquid interlayer or an eutectic liquid greatly enhances mass transport and bond formation [39]. Under applied pressure, part of the liquid eutectic phase may be expelled from the bonding zone. Subsequently, changes in the liquid phase composition can cause it to solidify at the bonding temperature, forming a continuous layer. However, if the thickness of this layer becomes excessive, a continuous layer of brittle intermetallic compounds may form during cooling, which is prone to cracking due to internal stresses [39]. This effect is further intensified by the significant difference in the coefficients of thermal expansion between copper and titanium, which leads to additional stresses during cooling and increases the likelihood of fracture in the interfacial region [102].

From the above, it follows that at temperatures above approximately 1093–1098 K, active growth of brittle intermetallic phases may occur in the Cu–Ti contact zone [46,100,101]. To achieve a combination of high plasticity and strength in Cu–Ti joints, the diffusion bonding temperature should be maintained closer to the lower limit of the 1073–1123 K range, while minimizing holding time and ensuring controlled, slow cooling.

Based on these considerations, the use of foil interlayers (e.g., Ni, Ti, Cu) or combined multilayer interlayers (e.g., Nb/Ti/Ni/Cu) is considered an effective approach to suppress or limit the formation of brittle intermetallic phases in the transition zone [103]. However, when joining composite multiphase materials rather than pure metals or simple alloys, selecting a suitable interlayer becomes more complex, as it is difficult to identify a material that provides optimal compatibility with all constituent phases [39,62,73].

Analysis of published studies [46,97] indicates that, in the Cu–Nb–M (M = Ni or Ti) ternary system, direct contact between specific metal pairs (e.g., Ti–Cu or Ni–Nb) generally leads to the formation of intermetallic compounds, making their formation difficult to avoid without the use of additional barrier layers.

To obtain a high-quality joint, it is necessary to limit both the bonding temperature and holding time, since at temperatures above approximately 1123 K, niobium fibers in Cu–Nb composites tend to coarsen, leading to a significant reduction in mechanical strength [43,104].

Nickel is widely considered an effective interlayer material for diffusion bonding between refractory metals and copper [63,64,103]. Nickel is also fully thermodynamically compatible with copper, but in the higher temperatures it reacts very actively with niobium, usually forming brittle compounds with it.

Although Ni offers advantages in terms of electrical conductivity, it was not used in the present study. Previous studies [63,64,103] indicate that Ni interlayers are applicable in such systems (Table 6); however, joints formed with Ni are typically more ductile but may exhibit lower strength compared to those formed using Ti interlayers.

Therefore, in this work, copper and titanium interlayers (commercially available foils with thicknesses of 0.15–0.4 mm) were employed in the bonding process, as Ti and Cu exhibit relatively high diffusion activity in contact with Cu–Nb composite materials (see Table 4) [75,80,101]. Further investigations involving interlayers with higher electrical conductivity (e.g., Ag, Ni and related materials) are ongoing, and the results will be reported in future work.

The selection of copper and titanium as intermediate layers was based on their complementary roles in the joining process, rather than on the assumption of complete thermodynamic compatibility. Copper was selected as a ductile and highly conductive interlayer that enhances the deformation of surface irregularities, improves physical contact under pressure, and minimizes electrical resistance loss in the joint. Titanium was selected as the reactive interlayer. The Ti–Nb interaction is favorable because Ti can form a solid solution with Nb, thereby promoting metallurgical bonding in the conductor regions containing Nb. However, at the Cu–Ti interface, titanium can form Cu–Ti intermetallic compounds and, at approximately 1123 K, a localized transitional liquid phase. This reactive behavior improves wetting and activate mass transfer, but also creates a risk of embrittlement and reduced electrical conductivity if a thick, continuous intermetallic layer forms. Therefore, the use of Ti requires careful control of the joining temperature, holding time, and Cu/Ti ratio. In this study, copper and titanium foils with thicknesses of 0.15–0.4 mm were combined to achieve a balance between interfacial activation, mechanical strength, and electrical conductivity [39,46,75,97,100,101].

Such interlayers in the diffusion bonding process mitigate thermal and mechanical mismatches. In this case, the copper foil interlayer reduces stress and, due to its lower yield strength compared to the Cu–Nb 18% microcomposite, promotes improved interfacial contact between the bonded surfaces under applied pressure.

**Figure 10:**
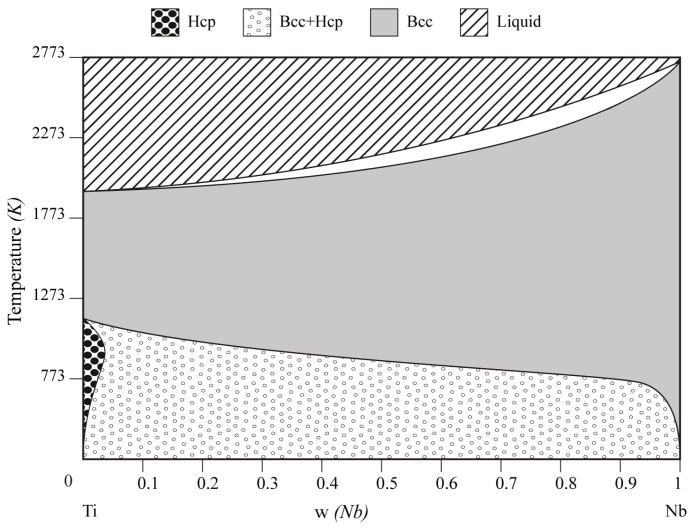
Ti-Nb Phase Diagram [46,108].

As shown for the Ti–Nb system (Figure 10), the use of Ti is justified by its extensive solubility in Nb, which promotes the formation of a stable metallurgical bond. Intensive interfacial reactions occur between the Ti interlayer and both substrates due to titanium’s high chemical reactivity.

**Figure 11:**
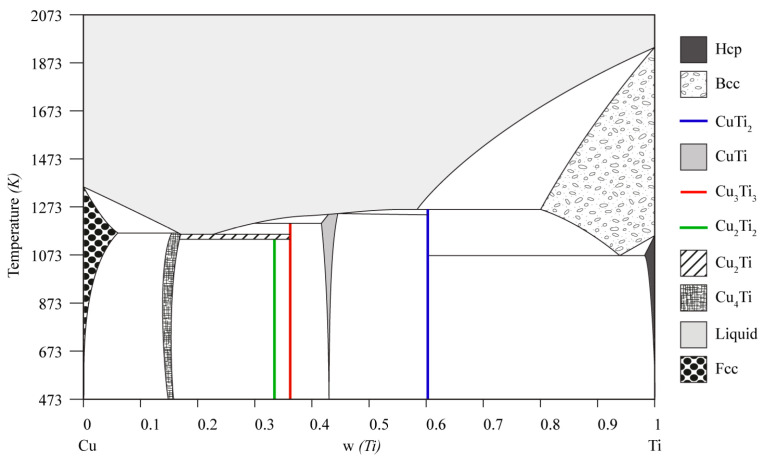
Cu-Ti Phase Diagram [46,68,72,109,110].

However, in this configuration, the Cu–Ti interface (Figure 11, Table 7) represents the most critical region. At temperatures above approximately 673 K, diffusion of Ti into Cu leads to the formation of brittle intermetallic compounds (Ti_2_Cu, TiCu, Ti_3_Cu_4_, Ti_2_Cu_3_, TiCu_4_) [39,105]. When the temperature exceeds approximately 1123 K, a transient liquid phase may form, significantly accelerating the growth of these compounds [105].

Excessive growth of the interfacial layer beyond approximately 5–10 μm results in pronounced embrittlement of the joint; therefore, it is necessary to minimize the time the joint is exposed to temperatures above 1073 K during the bonding process [106]. At lower temperatures (approximately 823–1073 K), the growth rate of intermetallic phases and the associated degradation of Nb fiber properties are significantly reduced [43,104,107].

**Table 7:** Invariant reactions in the Cu–Ti system [110].

T, K	Reaction
1262.0	Liquid + Bcc => CuTi_2_
1160.2	Liquid + Cu_4_Ti_3_ => Cu_2_Ti
1157.7	Liquid => Cu_4_Ti + Cu_2_Ti
1147.4	Cu_2_Ti + Cu_4_Ti_3_ => Cu_3_Ti_2_
1141.4	Cu_2_Ti => Cu_3_Ti_2_ + Cu_4_Ti
1071.4	Bcc => CuTi_2_ + Hcp

Note: BCC denotes a body-centered cubic solid solution, and HCP denotes a hexagonal close-packed solid solution. The listed reactions and temperatures represent equilibrium invariant reactions in the Cu–Ti system based on Ref. [110]. They are provided as a thermodynamic basis for interpreting the possible interfacial transformations and should not be regarded as direct experimental identification of individual phases in the investigated welded joints.

## 3. Diffusion Welding Equipment and Methodology of Welding Experiments

Prepared samples of the Cu–Nb conductor with polished ends (surface roughness Ra ≈ 1 µm) were placed in specially designed hollow frames made of a heat-resistant nickel-based alloy (ХН60ВТ) to ensure rigidity during welding. Immediately before welding, the Cu–Nb surfaces were degreased in alcohol using ultrasonic agitation, then etched in a solution containing 50 mL alcohol, 50 mL HF, and 30 mL HNO_3_. Diffusion bonding was performed in several configurations: with intermediate layers in various stacking combinations, and directly joining the conductor ends without interlayers.

It should be noted that the five joint configurations studied were produced using different diffusion welding systems. Series A–C were produced using glow-discharge diffusion welding, whereas series D and E were produced using a vacuum diffusion system with uniaxial pressure. Therefore, these results present an initial qualitative comparison of the investigated joints’ most important properties rather than a comprehensive assessment of all joints microstructure and phase transformations. In future phases of this research, a systematic multifactorial study will continue.

**Figure 12:**
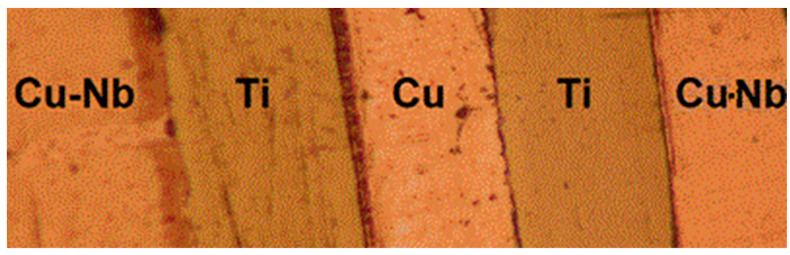
Example of Cu-Nb microcomposite joint foil assembly before welding.

Series A corresponds to joints produced using a Ti interlayer (0.4 mm Ti foil). Series B and C correspond to joints produced using Ti–Cu–Ti interlayer (0.15 mm Ti + 0.15 mm Cu + 0.15 mm Ti foils) (see Figure 12); and Cu–Ti–Cu interlayer (0.15 mm Cu + 0.15 mm Ti + 0.15 mm Cu foils), respectively. These three series were obtained using a glow discharge-assisted diffusion bonding system. Series D corresponds to joints produced using a Cu interlayer (0.2 mm Cu foil), whereas series E corresponds to butt joints produced without an interlayer. Series D and E were obtained using diffusion welding systems in vacuum.

Copper (99.9 wt.%) and titanium (99.9 wt.%) were used as the starting foil materials. The prepared joint components were placed in a press fixture within the diffusion bonding unit’s working chamber.

Although diffusion bonding offers significant advantages and unique capabilities, its application in European research and industry remains limited due to the restricted availability of specialized equipment. However, within the Erasmus mobility program for scientific and academic staff, the authors were able to conduct the planned experimental work.

Two specialized facilities at the National Technical University of Ukraine “Igor Sikorsky Kyiv Polytechnic Institute”, in cooperation with the E.O. Paton Institute of Materials Science and Welding, were used for joining the prepared samples.

Diffusion bonding was performed using a vacuum system UDV-35.01 (Tyazhelektrosvar, Kakhovka, Ukraine) equipped with electric resistance heating (through the welded components or external heaters) and an induction heating source, located at the E.O. Paton Electric Welding Institute. The system after modernization provides automatic operation, processes various alloys and dissimilar materials, and includes a vacuum chamber with integrated heating elements and a press unit. The working chamber volume is approximately 27 L, with a vacuum level below 10^−5^ mbar. The system enables heating up to 1573 K, with applied loads up to 50 kN and a heating zone height of 100 ± 5 mm. The welding time can reach 30 min.

The samples were heated using a radiation-based resistive heating system, and the temperature was monitored with platinum–rhodium Type S thermocouples suitable for high-vacuum operation.

Diffusion bonding in a glow discharge was performed using the ION-3 (E.O. Paton Electric Welding Institute, Kyiv, Ukraine) system, located at National Technical University of Ukraine “Igor Sikorsky Kyiv Polytechnic Institute”. The system enables automatic operation and processing of various alloys and dissimilar materials. It is equipped with a vacuum chamber incorporating resistive heating elements, a viewing window, and a press unit. The working chamber volume is approximately 30 L, with a vacuum level typically below 10^−5^ mbar. The system allows heating up to 1423 K, with applied loads up to 100 kN, a heating zone height of 200 ± 5 mm, and bonding times up to 60 min.

Temperature control was performed using platinum–rhodium Type S thermocouples suitable for high-vacuum operation. High-purity argon (Ar 6.0, group I1, EN ISO 14175 [111]) was used both for chamber venting and as the shielding gas during bonding. The chamber was initially evacuated to a pressure of approximately 100 Pa (1 mbar), followed by argon purging. The chamber was flushed with argon three times, and the working pressure during bonding was maintained at 19.6 kPa (147 Torr or 196 mbar).

Although the permissible ranges of welding parameters (energy input and time) were preliminarily estimated based on empirical relations and literature data, the final selection of welding modes was determined experimentally. The main variable parameters included heating temperature, applied force, and holding time.

Taking these considerations into account, the diffusion welding parameters were defined such that the bonding temperature did not exceed the melting temperature of copper (T_m_ = 1358 K) and was not lower than approximately 0.7 T_m_.

To evaluate the diffusion welding methodology and determine optimal processing conditions, an experimental program was developed. Five series of welding experiments were conducted using two diffusion welding systems: series A, B, and C were performed with the glow-discharge system, while series D and E were performed with the resistance-heated vacuum system.

In each series, specific welding parameters and interlayer configurations were varied. For series A, B, and C, the glow discharge voltage, applied pressure, and discharge duration were adjusted within the ranges required to achieve the target bonding temperatures under glow discharge and eutectic-assisted bonding conditions.

Series D and E focused on varying the diffusion bonding temperature, applied pressure, and holding time using conventional uniaxial pressing or soft interlayers.

**Figure 13:**
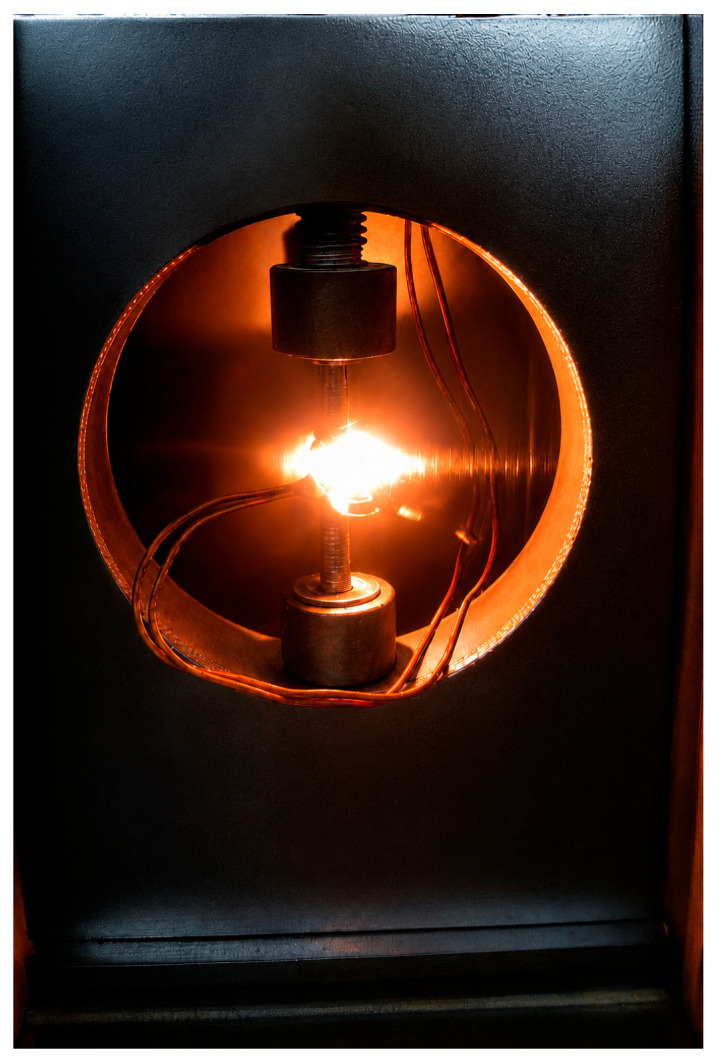
View of the process of wire diffusion welding with glow discharge (using the diffusion welding system ION-3).

Based on the results of the welding experiments, an initial assessment was conducted to evaluate the feasibility of bonding under the selected conditions, including the presence of defects, brittleness, and the mechanical integrity of the joints. The most suitable combinations of diffusion welding parameters were then selected for further analysis of the microstructure and properties of the resulting samples (Table 8 and Table 9; Figure 13).

The post-weld visual examination and preliminary evaluation of welding joints quality is performed using basic quality criteria. At this stage, the terms “acceptable,” “weak,” and “no bonding” refer only to the preliminary selection of specimens for further characterization and should not be interpreted as standards-based quality level, allowable defect levels and examination results according industrial standard requirements. A joint was classified as acceptable when a continuous bond was formed, the specimens remained connected after removal from the fixture and during routine handling, and no pronounced macroscopic defects, excessive deformation, significant extrusion of the interlayer material, visible bonding flaws, large melted eutectic regions were observed. A joint was classified as weak when bonding occurred but one or more of these defects were detected, making the specimen unsuitable for further detailed characterization. The term “no bonding” indicates that a continuous joint was not formed or that the specimen separated after removal from the fixture.

## 4. Methodology of Microstructural Analysis and Joints Properties Testing

### 4.1. Thermodynamic Calculations

In complex multiphase and heterogeneous materials, computational approaches such as CALPHAD (Calculation of Phase Diagrams) are widely used to calculate multicomponent phase equilibria and thermodynamic behavior. The CALPHAD method is based on the development of thermodynamic models describing individual phases, enabling the prediction of phase stability and properties in complex alloy systems. Several commercial software packages are available, including Thermo-Calc (Thermo-Calc Software AB, Stockholm, Sweden), FactSage (Thermfact Ltd., Montreal, QC, Canada), Pandat (CompuTherm LLC, Madison, WI, USA), MatCalc (MatCalc Engineering GmbH, Vienna, Austria) and JMatPro (Sente Software Ltd., Guildford, Surrey, UK), as well as open-source tools such as OpenCalphad, PyCalphad, and ESPEI [112,113,114].

Among these, Thermo-Calc is one of the most widely used thermodynamic software tools, providing access to numerous databases and enabling calculations of phase equilibria, phase compositions, transformation temperatures, and solubility limits. The DICTRA module, integrated with Thermo-Calc, allows for the simulation of diffusion-controlled transformations in multicomponent systems.

In the present study, Thermo-Calc software (version 2024b, Thermo-Calc Software AB, Stockholm, Sweden) was used to perform thermodynamic calculations of phase transformations using the TCCU6 database (TCS Copper-based Alloys Database) [115].

### 4.2. Metallographic Preparation and Microstructural Analysis

The morphology, microstructure and phase constituents of the diffusion-bonded joints were examined using scanning electron microscopy and optical microscopy. Butt-welded samples were cross-sectioned to evaluate the weld profile and microstructure. Specimens for microscopic investigation were prepared by abrasive grinding, diamond polishing, and subsequent electrolytic etching in a solution of 50 mL H_3_PO_4_ and 50 mL H_2_O. Electrolytic polishing and etching were performed using a PoliMat 2 (Buehler, IL, United States) system, with the samples immersed for up to 30 s at approximately 1 V.

The microstructure of diffusion-bonded Cu–Nb conductive joints was analyzed using an Axia™ ChemiSEM scanning electron microscope (Thermo Fisher Scientific, Waltham, MA, USA), equipped with an energy-dispersive X-ray spectroscopy (EDS) system for chemical microanalysis. SEM imaging was performed at accelerating voltages of 20 and 30 kV and a working distance of 10 mm in secondary electron (SE) mode.

The elemental composition and distribution of Cu, Nb, and Ti in the joint region were evaluated using microanalysis by energy-dispersive spectroscopy (EDS). Possible phase constituents were identified by comparing the EDS results with thermodynamic calculations performed using Thermo-Calc software version 2024b (Thermo-Calc Software AB, Stockholm, Sweden). Elemental mapping and line scanning by EDS were performed at accelerating voltages of 20 and 30 kV with a data acquisition time of 150 s.

Optical microscopy was performed using an Eclipse MA200 microscope (Nikon Corporation, Tokyo, Japan) equipped with a Moticam A16 digital camera (Motic, Xiamen, China).

### 4.3. Electrical Resistance Measurements and Thermal Imaging

The quality and properties of the welded conductor joints were evaluated in accordance with relevant standards, including GOST 10434 [60], EN 61788-6 [116], EN 61788-21 [117], and ISO 17279-3 [118].

In experimental practice, the four-point probe method, also known as the Kelvin method, is commonly used to determine the intrinsic electrical resistance of conductors [119]. This method is suitable for both bulk materials and thin films and provides high measurement accuracy for low-resistance samples.

The specific electrical resistance of the conductor was calculated according to Equation (17) [120,121,122].

(17) $$\rho =\frac{R\;S}{l}$$

where R—resistance of sample, Ω; S—cross-section area, m^2^; l—length of sample, m.

The electrical conductivity of a composite conductor can be calculated according to Equation (18) [120,121,122].

(18) $$\sigma =\frac{1}{\rho }$$

where σ—electrical conductivity (S m^−1^); ρ—specific electrical resistance (electrical resistivity) (Ω m).

Electrical resistivity measurements of samples were performed in accordance with ASTM B193-20 [123].

For electrical resistance measurements, three Cu–Nb wire specimens were prepared for each joint configuration. Each specimen was 70 mm long, with a distance of 32.59 mm between the measuring contacts (Table 10).

The experiments were conducted according to the methodology outlined in ISO 17279-3 [118]. Electrical resistance was measured using a U2810D digital LCR meter tester (EUCOL, Changzhou, China). Joule heating of the specimens due to electric current flow was monitored using a FLIR E49001 thermal imaging camera (FLIR Systems, Wilsonville, OR, USA). The samples were electrically loaded with a current of 200 A using a VDU-305 welding rectifier (Velga, Vilnius, Lithuania). The temperature distribution was recorded at the start of the experiment and at 30 s intervals thereafter.

### 4.4. Room-Temperature Tensile Testing

The key mechanical properties of the welded joints, including yield strength, ultimate tensile strength, and elongation, were determined by static tensile testing at room temperature in accordance with ISO 6892-1 [124], ISO 4136 [125], EN 61788-6 [116], and EN 61788-21 [117].

For this purpose, representative samples from each series were selected and welded using the selected preliminary processing conditions. A universal tensile testing machine 2055 P-0.5 (Tochpribor, Ivanovo, Russia), equipped with LabVIEW 2020 software (National Instruments, Austin, TX, USA) and PXI system hardware (NI PXIe-1073 chassis and NI PXIe-4330 controller, Austin, TX, USA), was used for testing. A Class 1.0 testing machine has a maximum permissible relative error of ±1.0% for indicated force and elongation. A 20 kN S-type tension load cell (Torbal, Bohemia, NY, USA) with a load measurement error of ±1.0% and a 3542 extensometer (Epsilon Technology, Jackson, WY, USA) with a gauge length of 25 mm were used for measurements.

Five unmachined specimens (Annex C, ISO 6892-1 [124]) from each series were tested. The distance between the grips of the testing machine was 40 mm, and the total length of the specimens with welded joints was 70 mm. The mechanical-property values reported in the corresponding tables represent the arithmetic mean of the five measurements.

### 4.5. High-Temperature Tensile Testing

The changes in the properties of the Cu–Nb microcomposite conductor at elevated temperatures were evaluated through high-temperature tensile tests. These data were used to estimate the required compression pressure during diffusion bonding, as the thermal conditions during testing are comparable to those encountered during bonding. The heating chamber of the tensile testing machine, equipped with resistive heating elements, was used to gradually increase the temperature to 500–1000 °C over approximately 0.5–1 h. These conditions closely simulate diffusion bonding, during which the microstructure degrades and the mechanical properties of the microcomposite conductor are reduced due to prolonged exposure to elevated temperatures.

High-temperature tensile testing was performed using an LFV-600-HH testing machine (Walter + Bai AG, Löhningen, Switzerland), class 1.0 according to ISO 7500-1 [126]. The system was equipped with a high-temperature furnace (STE-12 HR-350-SO, up to 1200 °C), a Eurotherm 3208 N temperature controller, a PCS 8000-T2 universal controller, and Dion 7 software. Testing was carried out in accordance with EN ISO 6892-2 [127].

A load cell (HBK, Hottinger Brüel & Kjær, Darmstadt, Germany) with a measurement error limit of ±0.5% (class 0.5 according to ISO 376) was used for force measurements. Strain was measured using a high-temperature extensometer Series 3448-050M-010 (Epsilon Technology, Jackson, WY, USA) with a gauge length of 50 mm and a strain measurement accuracy of 0.5% (class 0.5 according to ISO 9513).

**Figure 14:**
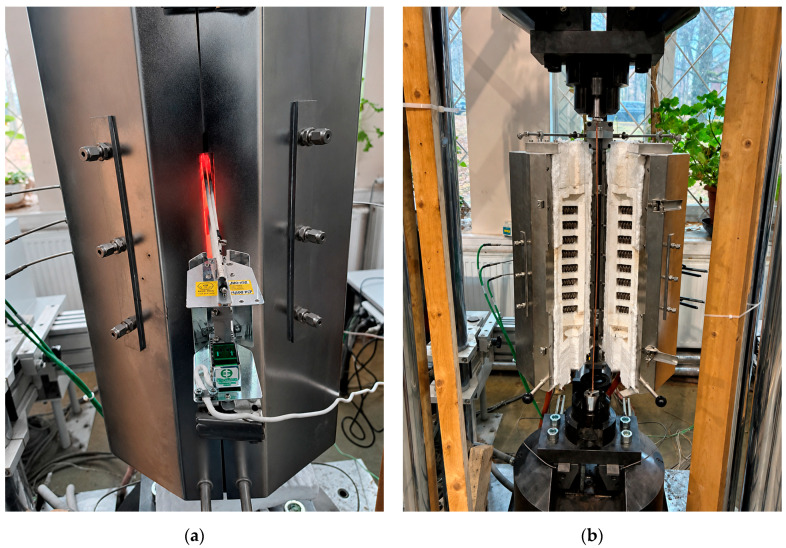
Walter Bai high-temperature tensile testing machine with heating chamber general view: (**a**) Description of what is contained in the first panel; (**b**) Description of what is contained in the second panel.

Five unmachined specimens (Annex A, ISO 6892-2 [127]) from each series were tested. The distance between the grips of the testing machine was 60 mm, and the total length of the specimens was 80 mm (Figure 14). The reported values represent the arithmetic mean of the five measurements. The force-measurement error limit was ±0.5%, and the strain-measurement accuracy was 0.5%.

## 5. Results and Discussion

### 5.1. Results of Simulation of Multicomponent Phase Behavior

**Figure 15:**
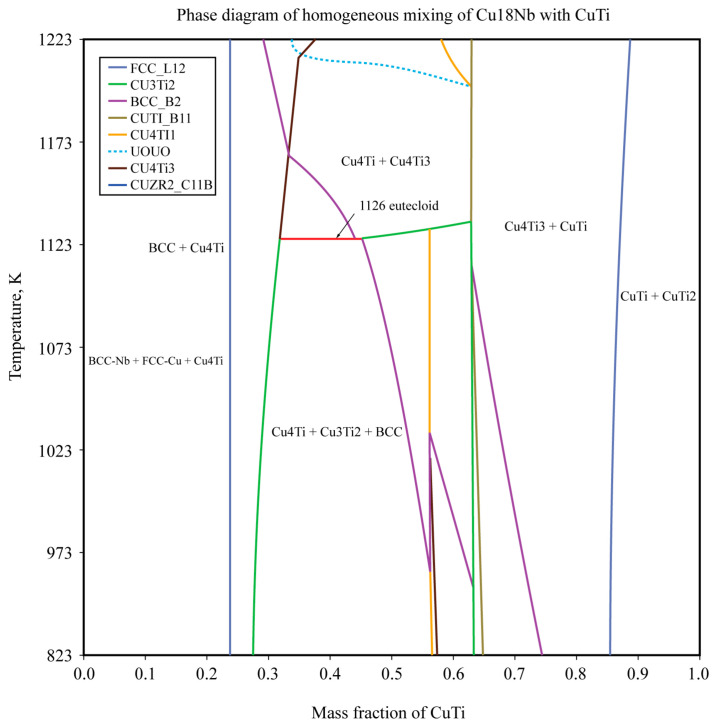
Phase diagram of homogeneous mixing of Cu-Nb conductor and Cu/Ti foils.

Analysis of the calculated ternary phase diagram using Thermo-Calc indicates that diffusion bonding of a Cu–Nb conductor with Cu and Ti foil interlayers requires a controlled proportion of Cu and Ti (with an approximate Cu/Ti ratio of 0.3–0.6) (Figure 15).

At lower proportions of Cu/Ti foils involved in mixing and diffusion bonding (up to approximately 0.25), and at bonding temperatures above 1126 K, the system consists of the BCC-Nb phase, the FCC-Cu phase, and the Cu_4_Ti intermetallic compound. This intermetallic phase is significantly harder and stronger than copper; however, it exhibits substantially lower electrical conductivity, approximately 20% IACS (with 100% IACS corresponding to 5.96 × 10^7^ S/m for copper) [128,129].

At higher titanium contents, a eutectic-type reaction occurs in the system at temperatures on the order of 1126 K. At elevated temperatures, the intermetallic compound Cu_4_Ti_3_ forms intensively. This phase, characterized by a tetragonal crystal structure, exhibits high hardness (approximately 2.6–2.8 GPa) and plays an important role in precipitation strengthening. However, it is typically associated with reduced electrical conductivity compared to pure copper [128,129].

**Figure 16:**
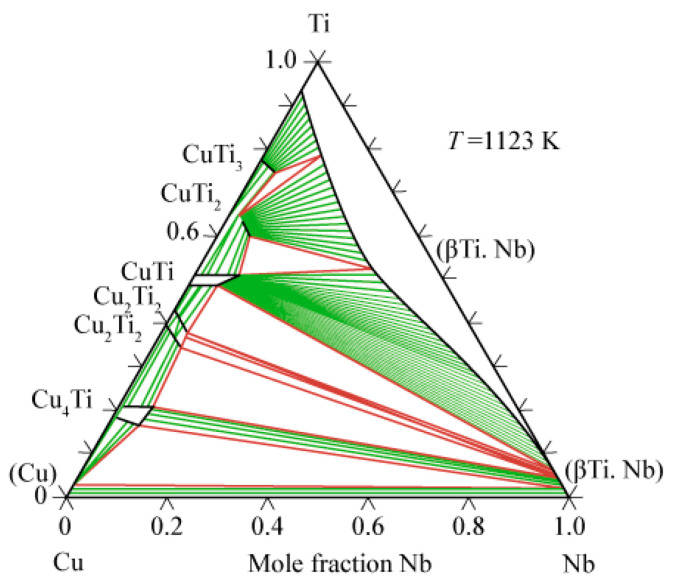
Calculated isothermal section of the Cu–Ti–Nb system at 1123 K, generated by using Thermo-Calc [46,97].

Thus, when using a titanium foil interlayer and a diffusion bonding temperature of approximately 1125 K, a thin eutectic liquid layer forms at the Cu–Ti interface due to contact melting between copper and titanium. This liquid phase enhances interfacial activation and, under the applied compressive load, redistributes along the interface before a thick, brittle intermetallic layer can form. As a result, a joint is formed according to the transient liquid phase (TLP) diffusion bonding mechanism (Figure 16) [130].

Thermodynamic calculations confirm that the selected interlayer system represents a compromise rather than an ideal combination. The Cu component improves electrical conductivity and plastic adaptation, whereas the Ti component promotes bond formation through reactive interactions and the local formation of a transitional liquid phase. At the same time, an excess of Ti or prolonged thermal exposure promotes the formation of brittle Cu–Ti intermetallic compounds with low conductivity. Therefore, the Cu/Ti ratio and the duration of bond formation must be controlled to limit the growth of a continuous layer enriched with intermetallic compounds.

The proposed bonding mechanism is based on the local formation of a transitional liquid phase and reactive wetting at the Cu–Ti interface. When heated to approximately 1123 K, the local composition at the interphase boundary can transition into a eutectic or liquid-containing phase, leading to the formation of a thin liquid layer. This phenomenon is known as contact melting or interface-induced liquefaction. This liquid phase promotes wetting, the dissolution of surface irregularities, and the accelerated redistribution of Cu and Ti. Under the action of an applied compressive load, part of the liquid phase may be displaced from the joint zone, while the remaining liquid changes its composition through mutual diffusion and subsequently solidifies upon cooling. The resulting transition zone contains Cu-enriched regions, Ti-containing solid solution regions, and discrete Cu–Ti intermetallic inclusions. The formation of a thick continuous intermetallic layer should be avoided, as this can lead to embrittlement. Under the conditions studied, the short reaction time and controlled Cu/Ti ratio limit the growth of a continuous layer enriched with intermetallic compounds.

Cu + Ti → localized Cu–Ti liquid-containing interfacial region → reactive wetting and dissolution of surface asperities → redistribution and partial expulsion of the liquid phase under pressure → solidification of a Cu-rich transition zone containing discrete Cu–Ti intermetallic inclusions and a thin localized intermetallic-rich layer.

### 5.2. Results of Calculations and Selection of Optimal Welding Parameters

In diffusion bonding processes, a diffusion layer thickness on the order of several micrometers (typically ~5–10 μm) is generally considered optimal. If the diffusion depth is too small (<1 μm) or excessively large (>15 μm), the joint may become brittle and susceptible to failure under thermal or mechanical loading [62].

From the calculated data presented in Table 11, it is evident that when Cu or Ti interlayers are used for diffusion bonding of Cu–Nb composite conductors, the required diffusion depth can be achieved only under a specific combination of bonding parameters and appropriate interlayer selection. The most suitable option appears to be a Cu interlayer, since at temperatures of 1125–1175 K the diffusion depth of Cu into the composite matrix, and vice versa, can reach approximately 5–10 μm. Comparable results can also be achieved using a Ti interlayer or a combination of Ti and Cu at 1075–1125 K.

The diffusion depths shown in Table 11 were calculated based on a solid-state diffusion model and should be interpreted as characteristic diffusion lengths under typical diffusion welding conditions. These calculated values are not directly equivalent to the entire width of the structurally modified transition zone present in joints produced by glow discharge. When titanium-containing interlayers are present, the local formation of a transient liquid phase and reactive wetting can significantly accelerate diffusion and increase diffusion depth. Therefore, the experimentally observed transition zone includes not only the solid-state diffusion layer but also the region of the intermediate layer and the zone affected by glow discharge.

The results of the welding experiments, conducted according to the developed experimental plan, enabled the selection of suitable bonding parameters for Cu–Nb conductors based on the quality of the joints. Based on the preliminary process analysis described above, the processing conditions indicated in Table 12 and Table 13 were selected for further microstructural, electrical, and mechanical characterization. These conditions should be considered as preliminary processing options rather than fully optimized parameters. Table 12 and Table 13 present the results of the welding experiments performed using the UDV-35.01 and ION-3 systems.

In the case of joints produced with a Cu interlayer, the joint quality is primarily influenced by the combination of bonding temperature in the range of 1125–1175 K (approximately 0.80–0.86 T_m_ of copper) and a holding time of 30–45 min. Lower temperatures resulted in incomplete bonding, whereas higher temperatures led to deterioration of joint quality, including the formation of interfacial voids and pronounced coarsening of Nb in the joint region. These defects contribute to embrittlement and reduced joint strength.

The required pressure and compressive force during welding (series D and E) were determined using Equation (9). Based on high-temperature mechanical test results and literature data, the pressure range was estimated to be 30–54 MPa. Forces exceeding approximately 400 N (corresponding to pressures above ~40 MPa) caused excessive deformation and significant extrusion of the interlayer material. A compression force of approximately 300 N was selected for further experiments, which did not exceed the wire material’s yield strength at the corresponding bonding temperature.

In the case of diffusion bonding using a glow discharge, the highest-quality joints were obtained at bonding temperatures in the range of 1075–1135 K (approximately 0.80–0.83 T_m_ of copper) and a holding time of 1–2 min. At lower temperatures, bonding did not occur, whereas at higher temperatures, joint quality deteriorated due to defect formation and partial melting.

The required welding pressure for samples (series A, B, and C) was determined using Equation (16). Based on high-temperature mechanical test results and literature data, the pressure range was estimated to be 3–6 MPa. Forces exceeding approximately 50 N (corresponding to pressures above ~5 MPa) resulted in excessive deformation and significant extrusion of the interlayer material. A compression force of approximately 50 N was selected for further experiments, which did not exceed the wire material’s yield strength at the corresponding bonding temperature.

In all cases, welded samples are cooled slowly at a rate of no more than 5–10 °K/min.

### 5.3. Results of Measurement of Electrical Characteristics of Connections

**Figure 17:**
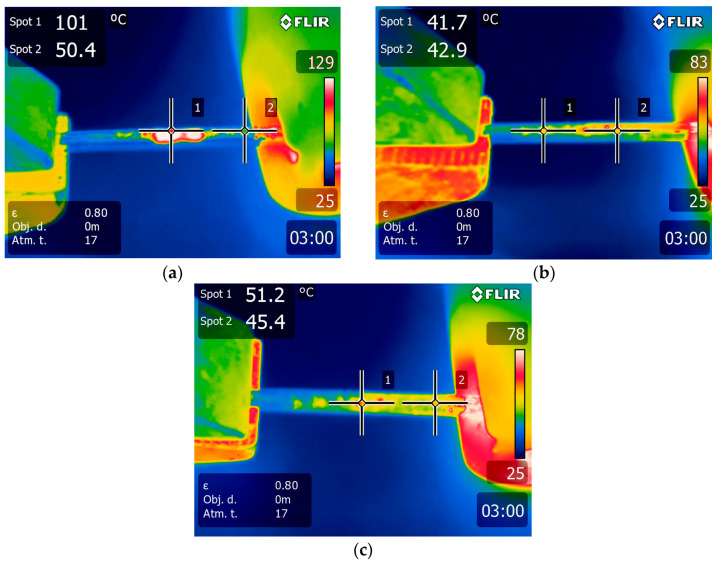
Temperature distribution in a diffusion weld: (**a**) the distribution of temperature in the specimen welded with Ti intermediate layer (A series) after 3 min of 200 A current flow; (**b**) the distribution of temperature in the specimen welded with Cu intermediate layer (D series) after 3 min of 200 A current flow; (**c**) the distribution of temperature in the specimen welded without intermediate layer (E series) after 3 min of 200 A current flow.

The thermal behavior of the welded joints under electrical loading was evaluated using a FLIR E49001 infrared thermal imaging camera (FLIR Systems, Wilsonville, OR, USA). The temperature distribution across the joint and the adjacent base material was monitored while a 200 A current was applied for 3 min. Representative thermal images recorded after 3 min of current flow are shown in Figure 17.

The maximum temperature observed in the weld zone of samples with a Ti interlayer (series A and B) reached approximately 374 K, while the base conductor remained significantly cooler (approximately two times lower) (Figure 17a).

In contrast, for samples produced using a Cu interlayer or by direct butt welding of the conductors (series D and E), the temperature distribution (Figure 17b,c) was nearly uniform. This behavior may be associated with the relatively high electrical conductivity of the joint region, particularly in the case of Cu interlayers.

The temperature difference between the diffusion bonding joint region and the base conductor during electrical current flow did not exceed the recommended limit of 368 K (according to GOST 17441) [131]. This indicates adequate thermal stability of the joints under Joule heating conditions. The results demonstrate that the diffusion-bonded joints can sustain high current loads without overheating or thermal degradation, confirming their suitability for pulsed power and other high-current applications.

It was established that the electrical conductivity of the Cu–18Nb microcomposite conductor and the corresponding diffusion-bonded joints is primarily influenced by the chemical composition of the interlayers, as well as the condition of the copper matrix, the distribution of niobium inclusions, and the extent of interphase boundaries (Table 14).

Among the welded joints studied, the samples joined with a copper interlayer exhibited the highest electrical conductivity, reaching 68.0% IACS. However, this value remained approximately 4.5% higher than the conductivity of the original Cu–Nb conductor (65.1% IACS). The relatively high conductivity of the joints with a copper interlayer may be primarily attributed to the high intrinsic conductivity of copper.

According to the information in [62], this effect can also be related to the fact that, in its initial heavily deformed state, copper contains a high density of defects and dislocations that scatter electrons. Upon heating to approximately 1123 K, recrystallization occurs, leading to a reduction in defect density, grain growth, and restoration of the crystal lattice. This results in a decrease in resistivity and an increase in conductivity by approximately 10–15% compared to the deformed state. However, no direct microstructural evidence of copper matrix recrystallization was obtained in this study. Indirect confirmation of our hypothesis may be the presence of a regular copper grain shape in the cross-section of the connection with a copper interlayer, which is not typical copper microstructure in foil production. Basically, this hypothesis and our interpretation should be considered preliminary and based on literature data.

The electrical conductivity of the specimens bonded without an interlayer is approximately 8.3% lower than that of the composite conductor. These results are consistent with observations reported for composite conductors subjected to butt resistance and flash welding. This behavior is likely associated with diffusion and coarsening of niobium at elevated temperatures. Fine Nb filaments, which initially act as barriers to dislocation motion, undergo fragmentation and spheroidization, forming chains of particles [132]. Although a reduction in the interfacial area between the Cu and Nb phases may decrease electron scattering, the accumulation of larger Nb particles at the joint interface increases electrical resistance, thereby reducing overall conductivity.

As expected, the use of Ti interlayers significantly reduces the electrical conductivity of the bonded specimens. However, the conductivity of joints produced with a Ti interlayer remains higher than that of pure titanium, which has relatively low electrical conductivity, typically in the range of 2–3.1% IACS [133].

At temperatures of approximately 1123 K, mutual diffusion occurs between copper and titanium [134]. Copper atoms diffuse toward the titanium interlayer; however, because copper is relatively soluble in titanium, the diffusion of titanium into copper plays a significant role in determining the electrical properties.

At elevated temperatures, titanium actively diffuses from the interlayer into the copper matrix. Even small concentrations of Ti in copper (solid solution) result in a significant increase in electrical resistivity due to lattice distortion and enhanced electron scattering [135].

In addition, the formation of intermetallic phases at the interface further increases electrical resistance, as these phases exhibit significantly lower electrical conductivity than pure copper. Upon cooling, the solubility of Ti in Cu decreases. Under slow-cooling conditions, partial precipitation of titanium from the supersaturated solid solution may occur, leading to a reduction in resistivity relative to the high-temperature state; however, the electrical conductivity remains lower than that of the initial material. Titanium may also interact with niobium to form a Ti–Nb solid solution [136]. However, this has a less pronounced effect on electrical resistance at room temperature compared to the influence of titanium in the copper matrix.

### 5.4. Results of Mechanical Properties Tests

Joints produced by diffusion bonding exhibited different mechanical and electrical properties depending on the interlayer type. The C series diffusion-welded samples with interlayers (Cu-Ti-Cu) exhibited the best mechanical properties compared with other welded samples. The maximum tensile strength achieved was 400 MPa, whereas samples manufactured by laser, electron beam, and magnetic pulse welding exhibited tensile strengths up to 360 MPa.

After long heating at a temperature of approximately 773 K, the conductor material loses about 15% of its mechanical strength. Prolonged keeping at this temperature (longer than 30 min) leads to the onset of copper recrystallization and irreversible changes in the morphology of niobium filaments. As a result, after cooling, the conductor’s strength does not recover to its original level of approximately 1120 MPa but instead stabilizes at around 950 MPa.

At temperatures of approximately 973 K during long heating (annealing conditions), fine niobium filaments undergo morphological transformation into cylindrical or spheroidal particles. As a result, the strength decreases to approximately 50–60% of the initial value.

The observed increase in ductility is associated with the release of internal stresses, as the stored deformation energy is consumed during recrystallization, and with an increased mean free path of dislocations. In the recrystallized copper matrix, dislocations can travel longer distances before interacting with niobium particles. This behavior indicates that the microcomposite gradually loses its characteristic filamentary strengthening mechanism and transitions toward a conventional dispersion-strengthened structure.

**Figure 18:**
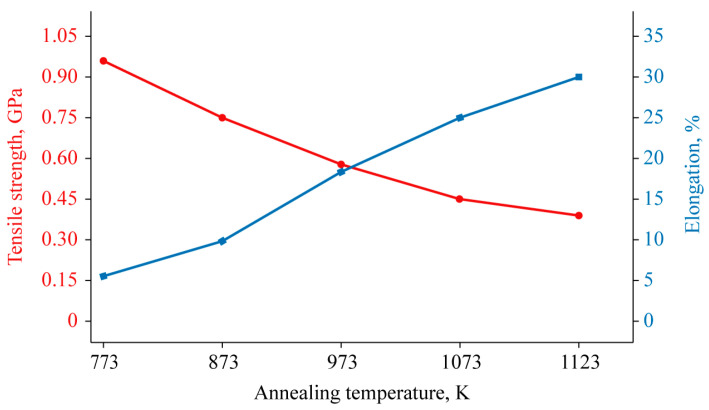
Effect of annealing temperature on the tensile strength and elongation of the Cu-Nb microcomposite conductor.

After approximately 30 min of annealing at a temperature above 1073 K, the tensile strength does not reach 400–450 MPa, which corresponds to the properties of copper reinforced by relatively coarse niobium particles (Table 15, Table 16 and Table 17, Figure 18). For clarity, the F-series designations used in Table 15 and Table 16 refer to the thermal heating conditions applied to the initial Cu–Nb conductor and should not be confused with the A–E series of diffusion-bonded joints.

### 5.5. Results of Welded Joints Microstructure Analysis

**Figure 19:**
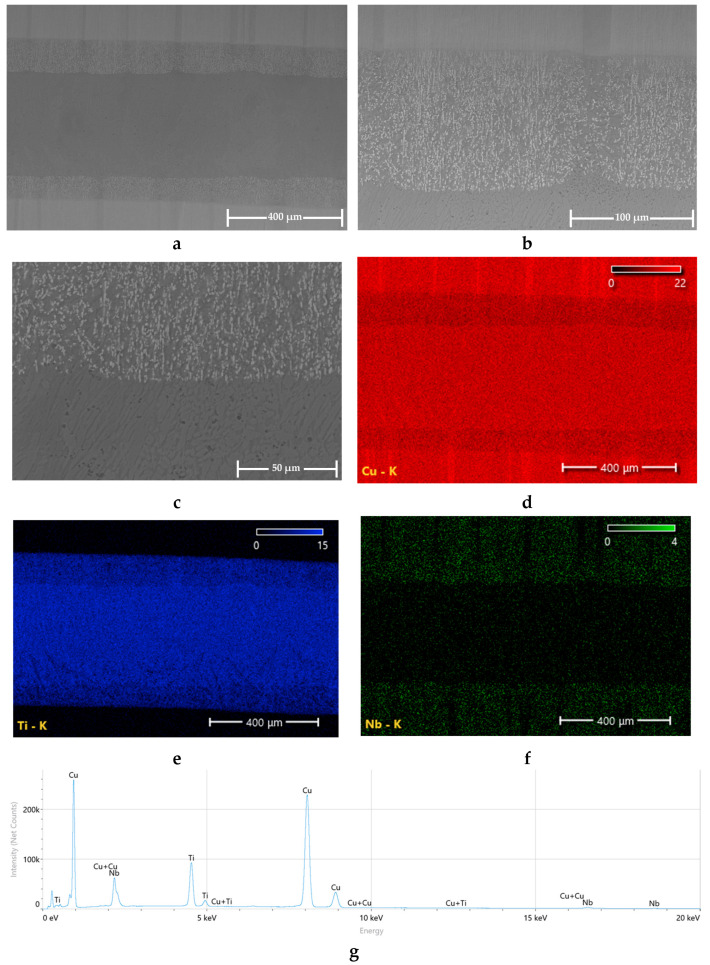
Diffusion weld with Ti interlayer (sample from series A): (**a**) ×350; (**b**) ×1500; (**c**) ×2500; (**d**) Cu elemental map; (**e**) Ti elemental map; (**f**) Nb elemental map; (**g**) EDS spectrum of the diffusion weld region containing Cu, Nb, and Ti.

**Figure 20:**
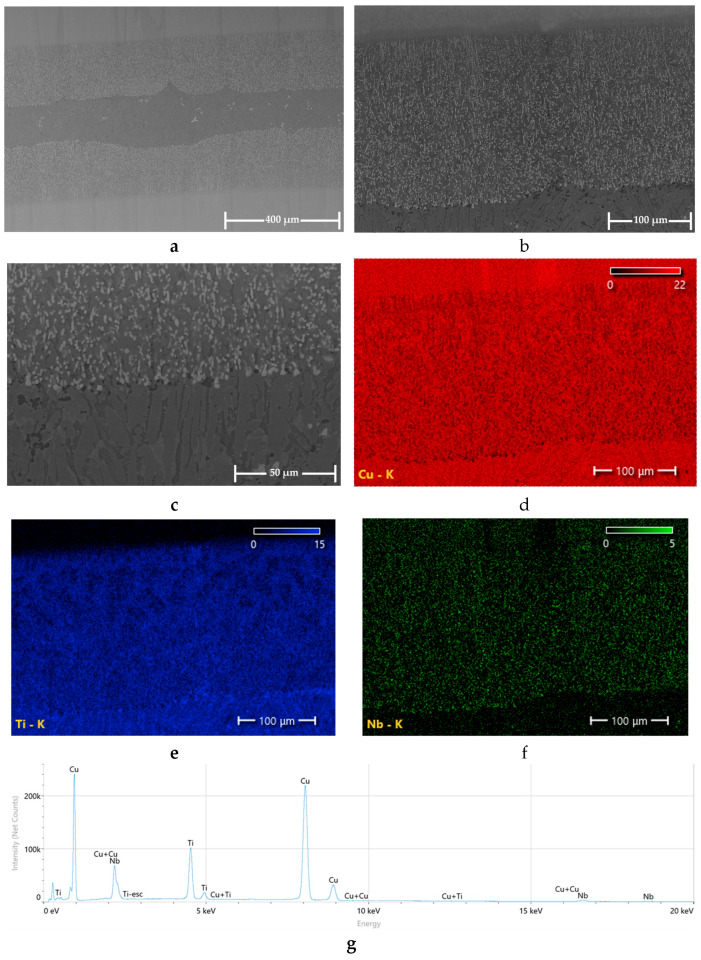
Diffusion weld with Cu and Ti interlayers (samples from series B and C): (**a**) ×350; (**b**) ×1000; (**c**) ×2500; (**d**) Cu elemental map; (**e**) Ti elemental map; (**f**) Nb elemental map; (**g**) EDS spectrum of the diffusion weld region containing Cu, Nb, and Ti.

**Figure 21:**
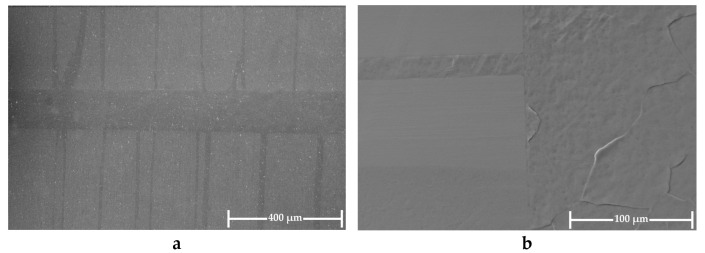

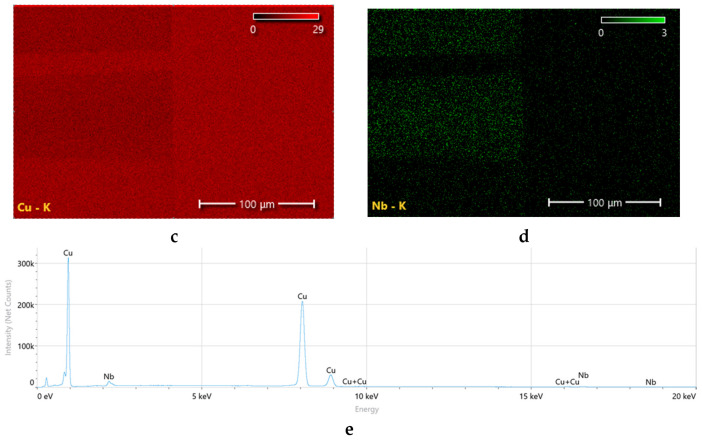
Diffusion weld with Cu interlayer (samples from series D): (**a**) ×350; (**b**) ×1500; (**c**) Cu elemental map; (**d**) Nb elemental map; (**e**) EDS spectrum of the diffusion weld region containing Cu and Nb.

**Figure 22:**
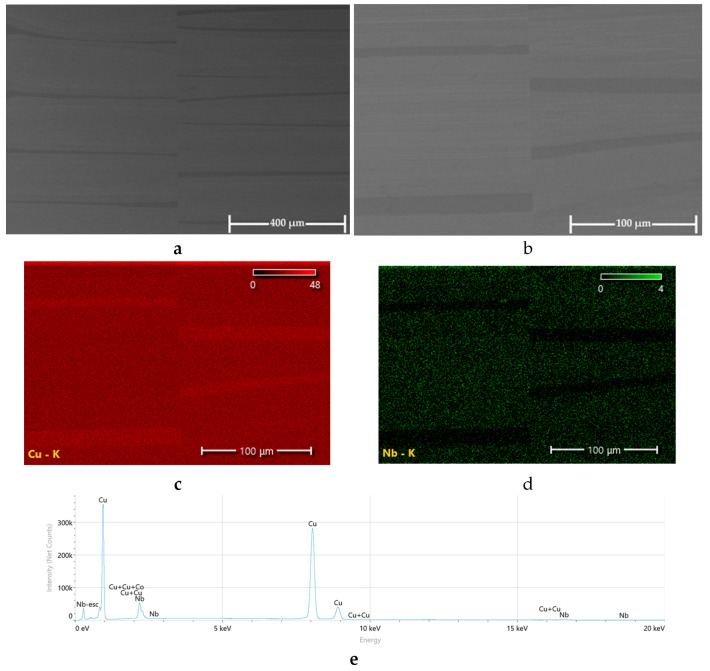
Diffusion weld without interlayer (sample from series E): (**a**) ×350; (**b**) ×1500; (**c**) Cu elemental map; (**d**) Nb elemental map; (**e**) EDS spectrum of the diffusion weld region containing Cu and Nb.

The microstructures of the cross-sections of the Cu–Nb conductor welded joints are shown in Figure 19, Figure 20, Figure 21 and Figure 22. As can be observed in Figure 19, in the welded joint of the Cu–Nb conductor using a Ti interlayer (series A), three distinct zones can be identified.

Zone 1 corresponds to the base conductor and exhibits no significant structural changes; the distribution of Cu and Nb elements remains characteristic of the initial microstructure. Zone 2 represents the interaction (diffusion) zone, where mutual diffusion of Ti and Cu occurs, accompanied by fragmentation and spheroidization of Nb filaments into particles with diameters of up to approximately 2 μm. The total fraction of inclusions (Nb and intermetallic compounds) in this zone is approximately 27% of the total area, and its width ranges from about 80 to 100 μm for diffusion welding using a glow discharge. The EDS spectrum (Figure 19g) indicates the presence of Cu–Ti phases, suggesting the formation of intermetallic compounds such as CuTi_2_, CuTi, Cu_4_Ti_3_, Cu_3_Ti_2_, and Cu_4_Ti, or eutectic structures formed during reactive wetting. Larger intermetallic inclusions (darker contrast), visible in Figure 19c, are localized near the interface with the Ti interlayer, while the thickness of the coarse intermetallic layer remains limited to approximately 5 μm to avoid embrittlement. Zone 3 corresponds to the Ti interlayer, into which Cu has diffused to a significant depth, resulting in the formation of a two-phase structure consisting of a solid solution (Cu with dissolved Ti) and intermetallic compounds. Discrete intermetallic inclusions (darker contrast), such as CuTi_2_, CuTi, Cu_4_Ti_3_, Cu_3_Ti_2_, and Cu_4_Ti, are observed in this zone. A characteristic variation in elemental concentration across the zones was confirmed by line-scan analysis (Table 18).

The microstructures of the cross-sections of the Cu–Nb conductor welded joints with combined Ti and Cu interlayers (series B and C) are shown in Figure 20, where three distinct zones can be identified. Zone 1 corresponds to the base conductor and exhibits no significant structural changes; the distribution of Cu and Nb elements remains characteristic of the initial microstructure. Zone 2 represents the interaction (diffusion) zone, where mutual diffusion of Ti and Cu occurs, accompanied by fragmentation and spheroidization of Nb filaments into particles with diameters reaching approximately 3–4 μm. The total fraction of inclusions (Nb and intermetallic compounds) in this zone is approximately 24% of the total area. The width of this zone is about 200 μm for diffusion welding using a glow discharge, which is comparable to the initial thickness of the Cu or Ti interlayers.

The EDS spectrum (Figure 20e) indicates the presence of Cu–Ti phases, suggesting the formation of a limited amount of intermetallic compounds such as CuTi_2_, CuTi, Cu_4_Ti_3_, Cu_3_Ti_2_, and Cu_4_Ti, as well as possible eutectic structures. Larger intermetallic inclusions (darker contrast), visible in Figure 20c, are localized near the interface with the Ti interlayer, while the thickness of the intermetallic-rich layer remains limited to approximately 5 μm, which is important for maintaining acceptable mechanical properties.

Zone 3 corresponds to the Ti interlayer, into which Cu has diffused throughout its thickness, resulting in the formation of a two-phase structure consisting of a solid solution (Cu with dissolved Ti) and intermetallic compounds. Discrete intermetallic inclusions (darker contrast), such as CuTi_2_, CuTi, Cu_4_Ti_3_, Cu_3_Ti_2_, and Cu_4_Ti, are observed in this zone. A notable feature of this joint is the absence of clearly distinguishable Cu interlayer regions. This can be explained by the rapid melting of the Cu interlayers during heating to approximately 1123 K, leading to the formation of a liquid phase (a low-melting eutectic or a solid solution of Cu and intermetallic compounds of Ti with Cu, such as Cu_2_Ti and Cu_4_Ti) upon contact between Cu and Ti. This is a classic example of a reactive wetting process. The resulting liquid phase actively interacts with the surrounding materials, dissolving the substrate and forming a transition layer. Since this process occurs within Zone 2, its width is approximately twice that observed when using only a Ti interlayer. A characteristic variation in elemental concentration across the zones was confirmed by line-scan analysis (Table 19).

It should be noted that in the experimental series A, B, and C, diffusion bonding with glow-discharge heating was employed, and all diffusion processes and phase transformations occurred within a relatively short time interval of 1–2 min.

The presence of Cu–Ti intermetallic compounds does not necessarily lead to an immediate loss of joint integrity. Their effect depends heavily on their morphology, continuity, and area thickness. Under the investigated glow discharge welding conditions, the region enriched with intermetallic compounds remained localized near the Ti-containing interface with a characteristic thickness of approximately 5 μm, whereas the remaining intermetallic compounds were observed mainly as discrete inclusions in the transition zone. The short welding time, approximately 1–2 min, limited the growth of a thick, continuous, brittle reaction layer. Consequently, the observed mechanical properties can be explained by the balance between liquid-phase-induced wetting, accelerated diffusion and mass transfer, on the one hand, and the limited growth of brittle Cu–Ti intermetallic compounds, on the other.

In the case of diffusion bonding of the Cu–Nb conductor using a Cu interlayer (series D), only two distinct zones can be identified. Zone 1 corresponds to the base conductor and exhibits no significant structural changes; the distribution of Cu and Nb elements remains characteristic of the initial microstructure. Zone 2 corresponds to the Cu interlayer region. In this case, no intermetallic compounds or eutectic phases are observed in the joint area (Figure 21), which is confirmed by EDS analysis (Figure 21e). Therefore, the strength of this joint is primarily governed by the self-diffusion of Cu, since Nb exhibits very low diffusivity. In addition, no noticeable coarsening or spheroidization of Nb is observed, in contrast to series A–C.

A similar behavior is observed in the case of diffusion butt bonding (series E) of the Cu–Nb microcomposite conductor. The joint is formed exclusively due to Cu diffusion, as only the interface line between the two conductor ends is visible in the cross-section (Figure 22), with no evidence of intermetallic inclusions. This is also confirmed by EDS analysis (Figure 22e).

High-resolution SEM imaging allows for a semi-quantitative assessment of the local morphological evolution of niobium filaments. The initial copper-niobium conductor contains fine niobium filaments with a diameter of less than 15 nm (what cannot be seen in presented SEM pictures due to the small magnification). In the longitudinal sections (Figure 19, Figure 20, Figure 21 and Figure 22) of the welded joints, only 200 μm diameter Nb filaments assemblies are visible, which in wire cross-section (Figure 4a) are visible in the form of hexagons.

In titanium-containing joints produced by glow discharge welding, local fragmentation and spheroidization of the niobium were observed in the interaction zone. For the configuration with a titanium interlayer (series A), the resulting niobium particles reached a diameter of approximately 2 μm, whereas for the combined configurations with a Ti/Cu interlayer (series B and C), the particle diameter reached approximately 3–4 μm. In contrast, in joints with a copper interlayer (Series D), no significant coarsening or spheroidization of niobium was observed under the investigated conditions. These observations indicate that diffusion bonding limits extensive structural damage associated with Nb coagulation, although, depending on the interlayer configuration and bonding conditions, local degradation of the original filamentary morphology may occur.

The comparison demonstrates that the calculated diffusion lengths and the experimentally observed intermetallic-compound layers are of similar magnitude, on the scale of several micrometers (Table 20). In the titanium-containing intermetallic compounds, the localized layer was enriched with intermetallic compounds and remained limited to approximately 5 μm. However, the total structurally modified interaction zone was significantly wider: approximately 80–100 μm for the configuration with a titanium interlayer and approximately 200 μm for combined configurations with titanium and copper interlayers. This difference indicates that the total width of the interaction zone is determined not only by solid-state diffusion. In the series obtained using a glow discharge, the local formation of a transient liquid phase, reactive wetting, redistribution of the interlayer material, and partial dissolution of the adjacent conductive regions contribute to the development of a wider transition zone. Accordingly, the calculated diffusion lengths should be regarded as first-order estimates of solid-state diffusion, rather than as direct predictions of the full width of the interaction zone.

## 6. Conclusions

Based on the results of the conducted studies, it can be concluded that Cu–Nb microcomposite conductors can be successfully joined by diffusion bonding to form a reliable permanent joint, although within a relatively narrow temperature range of 1075–1175 K. However, based on mechanical testing and microstructural analysis, it is more effective to use diffusion bonding with glow-discharge heating, as the short processing time (1–2 min) combined with the high intensity of the diffusion process results in significantly higher joint strength than conventional diffusion bonding. The results also indicate that diffusion bonding should be performed using interlayers, as direct joining of Cu–Nb microcomposite conductors does not provide sufficient joint strength or an adequate diffusion layer thickness. The use of intermediate layers that actively participate in diffusion and promote eutectic formation at interfaces enables stronger, permanent joints.

A key challenge in selecting suitable interlayer materials is ensuring not only adequate mechanical properties but also the high electrical conductivity of the joint. Experimental results obtained with titanium interlayers revealed that the main limitation of this approach is the reduced electrical conductivity of the joint. Nevertheless, the conducted work provides valuable insights. It establishes a clear direction for further research, particularly in the use of interlayer materials with higher electrical conductivity, such as nickel and silver.

## Figures and Tables

**Table 1:** Features of Diffusion Welding processes.

Type	Pressure Method	Heating Method	Key Feature	Best For
Hot Isostatic Pressing (HIP)	Isostatic (Gas)	Furnace	Uniform pressure on all surfaces	Complex shapes
Uniaxial Press Diffusion	Uniaxial (Mechanical)	Furnace/Resistance	Simple pressure application	Flat plates, simple geometries, clad metals
Eutectic/TLP Bonding	Uniaxial or Isostatic	Furnace	Uses a melting interlayer	Dissimilar materials, superalloys
Resistance (MFDC) Diffusion	Uniaxial	Resistance (Joule)	Fast, efficient, automated	High-volume production (connectors)
Vacuum Furnace Diffusion	Uniaxial	Radiant (Furnace)	Superior temperature and atmosphere control	Large parts, highest purity applications
Glow Discharge Diffusion	Uniaxial or Isostatic	Glow discharge plasma generation/high voltage	Controlled pressure application and atmosphere control in the chamber	Small parts

**Table 2:** Isobaric–isothermal potentials of oxidation reactions of selected metals under diffusion welding conditions in vacuum (0.7–0.8 T_m_, 10^−8^ atm) [23].

Metal	Reaction	Temperature, °K	Free Energy ΔG cal/mol in Vacuum 10^−8^	Oxidation Reaction
Cu	4Cu + O_2_ = 2Cu_2_O	1373	2040	Oxidation is unlikely or will not occur under the specified conditions
Cu	2Cu + O_2_ = 2CuO	1373	6000	Oxidation under these conditions is thermodynamically possible
Nb	4Nb + 5O_2_ = 2Nb_2_O_5_	2273	82,300	Oxidation is unlikely or will not occur under the specified conditions
Ti	Ti + O_2_ = TiO_2_	1773	106,400	Oxidation is unlikely or will not occur under the specified conditions

**Table 3:** Invariant reactions in the Cu-Nb system [47].

T, K	Reaction
1365.2	Liquid + body-centered cubic (BCC) structure => face-centered cubic (FCC)

Note: At 1365.2 K a liquid phase and a solid solution with a body-centered cubic (BCC) crystal structure react to form a solid solution with a face-centered cubic (FCC) crystal structure. BCC Phase—this refers to the body-centered cubic structure, which is the stable crystal form for Niobium (Nb) at these temperatures. FCC Phase—this refers to the face-centered cubic structure, which is the stable crystal form for pure Copper (Cu).

**Table 4:** Interface and self-diffusion coefficients of Cu, Nb, Ti couples in the solid state [75,79].

Diffusing Material	Host Material	Pre-Exponential Factor D_0_ (m^2^/s)	Activation Energy for Diffusion Q (kJ/mol)	Temperature Range T (°K)	Diffusion Coefficient at Temperature D (m^2^/s)
Nb	Nb	1.1 × 10^−4^	402	1075	3.2 × 10^−24^
Nb	Nb	1.1 × 10^−4^	402	1125	2.3 × 10^−23^
Nb	Nb	1.1 × 10^−4^	402	1175	1.5 × 10^−22^
Cu	Cu	7.8 × 10^−5^	210	1075	4.9 × 10^−15^
Cu	Cu	7.8 × 10^−5^	210	1125	1.4 × 10^−14^
Cu	Cu	7.8 × 10^−5^	210	1175	3.6 × 10^−14^
Nb	Cu	2 × 10^−4^	250	1075	1.4 × 10^−16^
Nb	Cu	2 × 10^−4^	250	1125	4.9 × 10^−16^
Nb	Cu	2 × 10^−4^	250	1175	1.5 × 10^−15^
Cu	Nb	1.5 × 10^−5^	231	1075	8.9 × 10^−17^
Cu	Nb	1.5 × 10^−5^	231	1125	2.8 × 10^−16^
Cu	Nb	1.5 × 10^−5^	231	1175	8 × 10^−16^
Ti	Cu	6.9 × 10^−5^	196	1075	2 × 10^−14^
Ti	Cu	6.9 × 10^−5^	196	1125	5.5 × 10^−14^
Ti	Cu	6.9 × 10^−5^	196	1175	1.3 × 10^−13^
Cu	Ti	3.8 × 10^−6^	190	1075	2.2 × 10^−15^
Cu	Ti	3.8 × 10^−6^	190	1125	5.7 × 10^−15^
Cu	Ti	3.8 × 10^−6^	190	1175	1.4 × 10^−14^
Ti	Nb	5.7 × 10^−6^	256	1075	2.1 × 10^−18^
Ti	Nb	5.7 × 10^−6^	256	1125	7.4 × 10^−18^
Ti	Nb	5.7 × 10^−6^	256	1175	2.4 × 10^−17^
Nb	Ti	2.1 × 10^−7^	189	1075	1.4 × 10^−16^
Nb	Ti	2.1 × 10^−7^	189	1125	3.5 × 10^−16^
Nb	Ti	2.1 × 10^−7^	189	1175	8.3 × 10^−16^

**Table 5:** Basic glow discharge diffusion welding parameters [90].

Parameter	Value
Current strength, A	10^−3^–10^2^
Anode voltage, V	up to 1000
Camera working pressure, kPa	10^−2^–10^2^
Parts compression force, MPa	up to 100
Maximum parts heating temperature, K	up to 2000
Current density at cathode, A/cm^2^	0.01–1

**Table 6:** Comparative table of properties of titanium and nickel substrates relative to the Cu-Nb pair.

Characteristics	Titanium (Ti)	Nickel (Ni)
Interaction with Nb	Ideal (unlimited solubility). No brittle phases are formed.	Limited. Brittle intermetallics (NbNi_3_) may form.
Interaction with Cu	Forms low-melting eutectics and a number of brittle intermetallics (Ti_2_Cu, TiCu, Ti_3_Cu_4_, Ti_2_Cu_3_, TiCu_4_)	Unlimited solubility. Forms a plastic solid solution. The most reliable contact with copper.
Role in a vacuum	Works as a “getter” (absorbs residual oxygen), purifying niobium.	Neutral, but requires better preliminary cleaning of surfaces.
Complexity of the procedure	High complexity. It is necessary to prevent the Ti + Cu zone from expanding.	Wide technological range in terms of time and temperature.

**Table 8:** Welding modes applied for samples diffusion welding on the UDV-35.01 system.

Intermediate Type	Temperature, K	Compression Force, N	Compression Pressure, MPa	Time, min	Bonding Quality
Option D					
D.1.1	1125–1135	400	40	45	Weak, bonding flaws
D.1.2	1175–1185	400	40	45	Weak, bonding flaws
D.1.3	1225–1235	400	40	45	Weak, Nb coagulation
D.2.1	1125–1135	400	40	30	Weak, bonding flaws
D.2.2	1175–1185	400	40	30	Weak, bonding flaws
D.2.3	1225–1235	400	40	30	Weak, Nb coagulation
D.3.1	1125–1135	400	40	15	Weak, bonding flaws
D.3.2	1175–1185	400	40	15	Weak, bonding flaws
D.3.3	1225–1235	400	40	15	Weak, bonding flaws
D.4.1	1125–1135	300	30	45	Acceptable
D.4.2	1175–1185	300	30	45	Weak, bonding flaws
D.4.3	1225–1235	300	30	45	Weak, Nb coagulation
D.5.1	1125–1135	300	30	30	Weak, bonding flaws
D.5.2	1175–1185	300	30	30	Acceptable
D.5.3	1225–1235	300	30	30	Weak, Nb coagulation
D.6.1	1125–1135	300	30	15	No bonding
D.6.2	1175–1185	300	30	15	Weak, bonding flaws
D.6.3	1225–1235	300	30	15	Weak, Nb coagulation
D.7.1	1125–1135	200	20	45	Weak, bonding flaws
D.7.2	1175–1185	200	20	45	Weak, bonding flaws
D.7.3	1225–1235	200	20	45	Weak, Nb coagulation
D.8.1	1125–1135	200	20	30	No bonding
D.8.2	1175–1185	200	20	30	Weak, bonding flaws
D.8.3	1225–1235	200	20	30	Weak, Nb coagulation
D.9.1	1125–1135	200	20	15	No bonding
D.9.2	1175–1185	200	20	15	No bonding
D.9.3	1225–1235	200	20	15	Weak, Nb coagulation
Option E					
E.1.1	1125–1135	400	40	45	Acceptable
E.1.2	1175–1185	400	40	45	Weak, bonding flaws
E.1.3	1225–1235	400	40	45	Weak, Nb coagulation
E.2.1	1125–1135	400	40	30	Weak, bonding flaws
E.2.2	1175–1185	400	40	30	Weak, bonding flaws
E.2.3	1225–1235	400	40	30	Weak, Nb coagulation
E.3.1	1125–1135	400	40	15	Weak, bonding flaws
E.3.2	1175–1185	400	40	15	Weak, bonding flaws
E.3.3	1225–1235	400	40	15	Weak, Nb coagulation
E.4.1	1125–1135	300	30	45	Weak, bonding flaws
E.4.2	1175–1185	300	30	45	Weak, bonding flaws
E.4.3	1225–1235	300	30	45	Weak, Nb coagulation
E.5.1	1125–1135	300	30	30	Weak, bonding flaws
E.5.2	1175–1185	300	30	30	Acceptable
E.5.3	1225–1235	300	30	30	Weak, Nb coagulation
E.6.1	1125–1135	300	30	15	No bonding
E.6.2	1175–1185	300	30	15	Weak, bonding flaws
E.6.3	1225–1235	300	30	15	Weak, Nb coagulation
E.7.1	1125–1135	200	20	45	Weak, bonding flaws
E.7.2	1175–1185	200	20	45	Weak, bonding flaws
E.7.3	1225–1235	200	20	45	Weak, Nb coagulation
E.8.1	1125–1135	200	20	30	No bonding
E.8.2	1175–1185	200	20	30	Weak, bonding flaws
E.8.3	1225–1235	200	20	30	Weak, Nb coagulation
E.9.1	1125–1135	200	20	15	No bonding
E.9.2	1175–1185	200	20	15	No bonding
E.9.3	1225–1235	200	20	15	Weak, Nb coagulation

Note: The bonding-quality categories refer to preliminary selection of specimens for further characterization. “Acceptable” indicates a joint selected for further characterization; “Weak” indicates a bonded specimen rejected because of visible defects or excessive deformation; “No bonding” indicates that a continuous joint was not formed.

**Table 9:** Diffusion welding modes for samples on the ION-3 installation.

Intermediate Type, Sample Number	Temperature, K	No-Load Voltage, V	Tension of the Glowing Discharge, V	Rushing Discharge Current, A	Compression Force, N	Compression Pressure, MPa	Time, min	Bonding Quality
A,B,C								
A,B,C 1.1	1075–1085	1000	200	0.6	70	7	1	No bonding
A,B,C 1.2	1125–1135	1000	210	0.6	70	7	1	Weak, bonding flaws
A,B,C 1.3	1165–1175	1000	220	0.6	70	7	1	Weak, large melted eutectic area
A,B,C 2.1	1075–1085	1000	200	0.6	70	7	2	Weak, bonding flaws
A,B,C 2.2	1125–1135	1000	210	0.6	70	7	2	Weak, bonding flaws
A,B,C 2.3	1165–1175	1000	220	0.6	70	7	2	Weak, large melted eutectic area
A,B,C 3.1	1075–1085	1000	200	0.6	50	5	1	No bonding
A,B,C 3.2	1125–1135	1000	210	0.6	50	5	1	Acceptable
A,B,C 3.3	1165–1175	1000	220	0.6	50	5	1	Weak, large melted eutectic area
A,B,C 4.1	1075–1085	1000	200	0.6	50	5	2	Acceptable
A,B,C 4.2	1125–1135	1000	210	0.6	50	5	2	Weak, bonding flaws
A,B,C 4.3	1165–1175	1000	220	0.6	50	5	2	Weak, large melted eutectic area
A,B,C 5.1	1075–1085	1000	200	0.6	30	3	1	No bonding
A,B,C 5.2	1125–1135	1000	210	0.6	30	3	1	Weak, bonding flaws
A,B,C 5.3	1165–1175	1000	220	0.6	30	3	1	Weak, large melted eutectic area
A,B,C 6.1	1075–1085	1000	200	0.6	30	3	2	No bonding
A,B,C 6.2	1125–1135	1000	210	0.6	30	3	2	Weak, bonding flaws
A,B,C 6.3	1165–1175	1000	220	0.6	30	3	2	Weak, large melted eutectic area

Note: The bonding-quality categories refer to preliminary selection of specimens for further characterization. “Acceptable” indicates a joint selected for further characterization; “Weak” indicates a bonded specimen rejected because of visible defects or excessive deformation; “No bonding” indicates that a continuous joint was not formed.

**Table 10:** Dimensions of conductor samples.

Parameters	Measurement Value
Length (l_1_)	70 mm
Height (a)	2.36 mm
Width (b)	4.2 mm
Cross-sectional area (A)	9.912 mm^2^
Distance between measurement clamps (l_2_)	32.59 mm

**Table 11:** Interface and self-diffusion coefficients of Cu, Nb, Ti couples in solid state.

Diffusion	Temperature T (K)	Diffusion Coefficient at Temperature D (m^2^/s)	Diffusion Depth X, (μm) at Diffusion Time T = 1800 (s)	Diffusion Depth X, (μm) at Diffusion Time T = 2700 (s)	Diffusion Depth X, (μm) at Diffusion Time T = 3600 (s)
Nb→Nb	1075	3.2 × 10^−24^	7.6 × 10^−5^	9.3 × 10^−5^	1.1 × 10^−4^
Nb→Nb	1125	2.3 × 10^−23^	2 × 10^−4^	2.5 × 10^−4^	2.9 × 10^−4^
Nb→Nb	1175	1.5 × 10^−22^	5.2 × 10^−4^	6.4 × 10^−4^	7.4 × 10^−4^
Cu→Cu	1075	4.9 × 10^−15^	3	3.7	4.2
Cu→Cu	1125	1.4 × 10^−14^	5	6.2	7.1
Cu→Cu	1175	3.6 × 10^−14^	8	9.9	11.4
Nb→Cu	1075	1.4 × 10^−16^	0.5	0.6	0.7
Nb→Cu	1125	4.9 × 10^−16^	0.9	1.2	1.3
Nb→Cu	1175	1.5 × 10^−15^	1.6	2	2.3
Cu→Nb	1075	8.9 × 10^−17^	0.4	0.5	0.6
Cu→Nb	1125	2.8 × 10^−16^	0.7	0.9	1
Cu→Nb	1175	8 × 10^−16^	1.2	1.5	1.7
Ti→Cu	1075	2 × 10^−14^	6	7.3	8.5
Ti→Cu	1125	5.5 × 10^−14^	10	12.2	14.1
Ti→Cu	1175	1.3 × 10^−13^	15.3	18.8	21.6
Cu→Ti	1075	2.2 × 10^−15^	2	2.4	2.8
Cu→Ti	1125	5.7 × 10^−15^	3.2	3.9	4.5
Cu→Ti	1175	1.4 × 10^−14^	5	6.2	7.1
Ti→Nb	1075	2.1 × 10^−18^	6.1 × 10^−2^	7.5 × 10^−2^	8.7 × 10^−2^
Ti→Nb	1125	7.4 × 10^−18^	1.1 × 10^−1^	1.4 × 10^−1^	1.6 × 10^−1^
Ti→Nb	1175	2.4 × 10^−17^	0.2	0.25	0.3
Nb→Ti	1075	1.4 × 10^−16^	0.5	0.6	0.7
Nb→Ti	1125	3.5 × 10^−16^	0.79	0.97	1.12
Nb→Ti	1175	8.3 × 10^−16^	1.2	1.5	1.7

**Table 12:** Selected diffusion welding modes for samples on the UDV-35.01 installation.

Interlayer Type	Temperature, K	Compression Force, N	Compression Pressure, MPa	Time, min
Option D (Cu)				
D.4.1	1125–1135	300	30	45
D.5.2	1175–1185	300	30	30
Option E (without interlayer)				
E.1.1	1125–1135	400	40	45
E.5.2	1175–1185	300	30	30

**Table 13:** Selected diffusion welding modes for samples on the ION-3 installation.

Intermediate Type, Sample Number	Temperature, K	No-Load Voltage, V	Tension of the Glowing Discharge, V	Rushing Discharge Current, A	Compression Force, N	Compression Pressure, MPa	Time, min
A,B,C 4.1	1075–1085	1000	210	0.6	50	5	2
A,B,C 3.2	1125–1135	1000	210	0.6	50	5	1

**Table 14:** Results of electrical resistance and electrical conductivity measurements of samples (measurements using the 4-point method).

Sample	Electrical Resistance of Sample, Ω	Specific Electrical Resistance According to Equation (17) Ω m	Electrical Conductivity, S m^−1^	Electrical Conductivity σ (IACS %)
Conductor	8.708 10^−5^	2.648 10^−8^	3.776 10^7^	65.10
A series	4.724 10^−4^	1.437 10^−7^	6.960 10^6^	12.00
B series	2.834 10^−4^	8.619 10^−8^	1.160 10^7^	20.00
C series	1.099 10^−4^	3.343 10^−8^	2.992 10^7^	51.60
D series	8.337 10^−5^	2.536 10^−8^	3.944 10^7^	68.00
E series	9.496 10^−5^	2.888 10^−8^	3.462 10^7^	59.70

**Table 15:** Results of high-temperature tensile tests of Cu-Nb wires at different heating temperatures.

Conductor Sample	Heating Temperature, (K)	Heating Time, (min)	Yield Strength, (GPa)	Tensile Strength, (GPa)	Elongation, (%)
F1 series	773	30	0.38	0.7	9
F2 series	873	40	0.2	0.48	14
F3 series	973	50	0.12	0.31	25
F5 series	1073	60	0.09	0.20	34
F6 series	1123	65	0.06	0.15	39

Note: The reported values are arithmetic means based on five specimens tested for each condition or joint configuration. Testing machine is calibrated and classified by maximum permissible relative error (Class 1.0: ±1.0%) used for indicated force and elongation measurement.

**Table 16:** Results of room temperature tensile tests of annealed Cu-Nb wires.

Conductor Sample	Annealing Temperature, (K)	Annealing Time, (min)	Yield Strength, (GPa)	Tensile Strength, (GPa)	Elongation, (%)	Microstructure
F1 series	773	30	0.74	0.95	6	Start of Cu recrystallization
F2 series	873	30	0.63	0.75	10	Intensive recrystallization of Cu
F3 series	973	30	0.43	0.58	18	Degradation of Nb fibers
F5 series	1073	30	0.3	0.45	25	Spheroidization of Nb fibers
F6 series	1123	30	0.25	0.4	30	Coagulation of Nb

Note: The reported values are arithmetic means based on five specimens tested for each condition or joint configuration.

**Table 17:** Results of room temperature tensile tests of samples with diffusion-welded joints.

Welded Sample with an Interlayer	Yield Strength, (GPa)	Tensile Strength, (GPa)	Elongation, (%)
Ti	0.17	0.33	1.5
Ti-Cu-Ti	0.2	0.37	2.0
Cu-Ti-Cu	0.22	0.4	2.5
Cu	0.12	0.26	0.6
Without interlayer	0.15	0.3	0.5

Note: The reported values are arithmetic means based on five specimens tested for each condition or joint configuration.

**Table 18:** Distribution and concentration of elements in different zones of the welded joint.

Element	Concentration of Element, wt % (in 1 Area)	Concentration of Element, wt % (in 2 Area)	Concentration of Element, wt % (on the Edge of 3 Area)
Cu	82	74	88
Ti	0	8	12
Nb	18	18	0

**Table 19:** Elemental distribution and concentration in different zones of the welded joint.

Element	Concentration of Element, wt % (in 1 Area)	Concentration of Element, wt % (in 2 Area)	Concentration of Element, wt % (on the Edge of 3 Area)
Cu	82	77	93
Ti	0	5	7
Nb	18	18	0

**Table 20:** Comparison of calculated diffusion lengths and experimentally observed interaction-zone characteristics for the investigated joints.

Joint Configuration	Bonding Mechanism	Calculated Characteristic Diffusion Length	Experimentally Observed Feature	Interpretation
Cu interlayer, series D	Predominantly solid-state Cu diffusion	Approximately 5–10 μm under the selected conventional bonding conditions	No intermetallic compounds or eutectic phases were detected; the joint region is associated primarily with the Cu interlayer thickness	The solid-state model is applicable as a first approximation
Without interlayer, series E	Predominantly solid-state Cu diffusion	Limited by low Cu–Nb interdiffusion	Only the interface line is visible; no intermetallic inclusions were detected	Limited diffusion explains the lower bonding efficiency
Ti interlayer, series A	Reactive interaction with possible localized transient liquid phase	Solid-state diffusion lengths are on the order of several micrometers	Intermetallic-rich layer: approximately 5 μm; total modified interaction zone: approximately 80–100 μm	The narrow intermetallic-rich layer is consistent with the calculated micrometer-scale diffusion length; the broader zone reflects reactive wetting and redistribution
Ti–Cu–Ti and Cu–Ti–Cu interlayers, series B–C	Reactive wetting and accelerated diffusion	Solid-state diffusion lengths are on the order of several micrometers	Intermetallic-rich layer: approximately 5 μm; total modified interaction zone: approximately 200 μm	The broader zone cannot be explained by solid-state diffusion alone and is associated with interlayer redistribution and transient-liquid-phase-assisted interaction

## Data Availability

The original contributions presented in the study are included in the article; further inquiries can be directed to the corresponding author.

## Abbreviations

The following abbreviations are used in this manuscript:

DFW	Diffusion welding
TLP	Transient liquid phase
MFDC	Mid-frequency direct current bonding/resistance-based bonding
BCC	Body-centered cubic structure
FCC	Face-centered cubic structure
IACS	International Annealed Copper Standard
TRIZ	Theory of Inventive Problem Solving
CALPHAD	Calculation of Phase Diagrams
SEM	Scanning electron microscope
EDS	Energy Dispersive X-ray Spectroscopy
HIP	Hot isostatic pressing
